# Identifying genes associated with genetic control of color polymorphism in the pearl oyster *Pinctada margaritifera* var. *cumingii* (Linnaeus 1758) using a comparative whole genome pool‐sequencing approach

**DOI:** 10.1111/eva.13464

**Published:** 2022-09-16

**Authors:** Pierre‐Louis Stenger, Chin‐Long Ky, Jeremie Vidal‐Dupiol, Serge Planes, Céline Reisser

**Affiliations:** ^1^ Ifremer, IRD, Institut Louis‐Malardé Univ Polynésie française, EIO Vairao France; ^2^ IHPE, Univ. Montpellier, CNRS, Ifremer, Univ. Perpignan Via Domitia Montpellier France; ^3^ PSL Research University, EPHE‐UPVD‐CNRS, USR 3278 CRIOBE, Labex Corail, Université de Perpignan Perpignan Cedex France; ^4^ MARBEC, Univ Montpellier, CNRS, IFREMER, IRD Montpellier France

**Keywords:** color, pearl farming, pigmentation evolution, *Pinctada margaritifera*, pooled whole genome sequencing, population genomics

## Abstract

For hundreds of years, the color diversity of Mollusca shells has been a topic of interest for humanity. However, the genetic control underlying color expression is still poorly understood in mollusks. The pearl oyster *Pinctada margaritifera* is increasingly becoming a biological model to study this process due to its ability to produce a large range of colors. Previous breeding experiments demonstrated that color phenotypes were partly under genetic control, and while a few genes were found in comparative transcriptomics and epigenetic experiments, genetic variants associated with the phenotypes have not yet been investigated. Here, we used a pooled‐sequencing approach on 172 individuals to investigate color‐associated variants on three color phenotypes of economic interest for pearl farming, in three wild and one hatchery populations. While our results uncovered SNPs targeting pigment‐related genes already identified in previous studies, such as PBGD, tyrosinases, GST, or FECH, we also identified new color‐related genes occurring in the same pathways, like CYP4F8, CYP3A4, and CYP2R1. Moreover, we identified new genes involved in novel pathways unknown to be involved in shell coloration for *P. margaritifera*, like the carotenoid pathway, BCO1. These findings are essential to possibly implement future breeding programs focused on individual selection for specific color production in pearl oysters and improve the footprint of perliculture on the Polynesian lagoon by producing less but with a better quality.

## 1 INTRODUCTION

The color diversity of Mollusca shells has been a topic of interest for scientists since hundreds of years (Williams, 2016). Mollusca is the largest phylum in the marine ecosystem, which explains that, ultimately, only a small portion of its species has been studied. Consequently, the evolution of their widely diverse colors and shapes is poorly understood. Species with economical interest have historically been used by scientists to understand fundamental biological mechanisms such as color variation in bivalves. Marine bivalves, like the scallops *Patinopecten yessoensis* (Jay, 1857) (Chang et al., 2007; Ding et al., 2015; Sun et al., 2015; Yuan et al., 2021), *Argopecten irradians* (Lamarck, 1819) (Adamkewicz & Castgna, 1988; Du et al., 2017), or the Pacific oyster *Crassostrea gigas* (Thunberg, 1793) (Aguilera et al., 2014; Bonnard et al., 2020, 2021; Feng et al., 2015, 2018; Hu et al., 2021; Song et al., 2017) have already received much attention for the elucidation of their pigmentation pathways. More recently, the black‐lipped pearl oyster *Pinctada margaritifera* var. *cumingii* (Linnaeus, 1758) emerged as a model to study pigmentation pathways (Ky et al., 2016, 2018; Ky, Le Pabic, et al., 2017; Stenger et al., 2019; Stenger, Ky, Reisser, Cosseau, et al., 2021; Stenger, Ky, Reisser, Duboisset, et al., 2021). While color has been studied in scallops and oysters because of the importance of their appearance and visual flesh and shell quality for the food industry (Ding et al., 2015), the color of the black‐lipped pearl oyster inner shell is of importance for pearl production. *P. margaritifera* has the remarkable ability to produce one of the largest ranges of colored pearls for a marine species (Ky et al., 2014). This makes the Tahitian pearls unique and places pearl farming as the second economic resource in French Polynesia (Bouzerand, 2018). However, the lack of knowledge about how pearl quality is determined in the oyster, how it can be controlled, and an increasing occurrence of bad quality pearls on the market has led to the collapse of the pearl's economic value since 2001 (Bouzerand, 2018). To remedy the crisis, a new policy aiming at producing less while increasing the quality has been enforced by local authorities, and research programs on the study of pearl quality trait determination and control began (Latchere et al., 2018; Stenger et al., 2019).

Traditional approaches to identify the proportion of genetic control of a trait involves experimental crossings between phenotypes (Zheng et al., 2013) and self‐fertilized mattings (Adamkewicz & Castgna, 1988) (or a combination of both techniques) (Kobayashi et al., 2004) to produce F1 and F2 generations and observe the segregation of a trait of interest while making assumptions about the underlying genetic control. These techniques have been used for the bluish versus greenish strains of the Pacific abalone *Haliotis discus hannai* (Kobayashi et al., 2004), and in the orange versus yellow bay scallops *A. irradians* strains (Adamkewicz & Castgna, 1988), for which the shell color is determined by a similar one‐locus two‐allele system in both species. In the Chilean scallop *Argopecten purpuratus*, the purple, brown, orange, yellow, and white color strains were determined by two loci with a simple dominant model of epistasis (Winkler et al., 2001). In the noble scallop *Chlamys nobilis* (Reeve, 1852), the orange–purple, purple, and brown colors are explained by a one‐locus three‐alleles model (Zheng et al., 2013). In the black‐lipped pearl oyster *P. margaritifera*, a “one‐locus three‐alleles” model with dominance was proposed to control external shell color, whereby the black allele is dominant to the red allele, which itself is dominant to the white “albino” allele (Ky et al., 2016). For the inner color of the shell, experimental crosses between red males and red females resulted in close to 100% red F1 (Ky et al., 2016), whereas crosses between yellow males and yellow females or between green males and green females gave both around 50% of yellow and 50% of green F1 (C.‐L. Ky, personal communication). These results suggest that the red phenotype is likely controlled by a few genes, with high impact alleles, whereas the control of yellow and green phenotype likely depends on multiple genes with low impact alleles (polygenic). Recently, transcriptomic (Stenger, Ky, Reisser, Duboisset, et al., 2021) and epigenomic (Stenger, Ky, Reisser, Cosseau, et al., 2021) studies have for the first time identified pathways leading to color variability in this species, and some key genes. However, putative genomic variants that could control those phenotypes have not yet been described.

Genetic variants involved in color phenotypes have been extensively studied in vertebrates. In felines, the acquisition of a stop codon in the tyrosinase‐related protein 1 (TYRP1) gene through a substitution from T to C at position 298 leads to a cinnamon phenotype (light brown color coat) (Lyons et al., 2005). A missense mutation is responsible for the replacement of the 618 arginines with a cysteine in the premelanosome protein 17 (PMEL17) gene, changing its 3D conformation and leading to the silver coat color (the uncommon silver dapple phenotype) in horses (Brunberg et al., 2006). SNPs that are located directly upstream, downstream, or within intronic regions can also control color phenotypes by modulating gene expression. According to Hart et al. (2013), an upstream SNP of the Solute Carrier Family 2 Member 4 gene (SLC2A4) produces blue eyes and light skin in humans (Hart et al., 2013). A single SNP in the intron of the HECT and RLD Domain‐Containing E3 Ubiquitin Protein Ligase 2 gene (HERC2) at position 86 determines the human blue‐brown eye color (Sturm et al., 2008). Several other studies have also demonstrated that a single SNP in an intron can modify the expression of neighboring genes (Shima et al., 2006; Tokuhiro et al., 2003). Moreover, SNPs contained in intergenic regions can also impact phenotypic traits through their proximity to long intergenic noncoding RNAs (LincRNA) (Hangauer et al., 2013). In the Pacific oyster *Crassotrea gigas*, LincRNAs affect the expression of the pigment‐related genes tyrosinase‐like proteins, dopamine, β‐monooxygenase, chorion peroxidase, and cytochrome P450 2 U1, leading to different color phenotypes (Feng et al., 2018).

Since the rise of next‐generation sequencing in the last decade, it is now possible to sequence entire genomes and identify color‐associated SNPs. Genome‐wide association studies with shell coloration were carried out for *Cepaea nemoralis* (Linnaeus, 1758) (Richards et al., 2013), *P. yessoensis* (Ding et al., 2015), and *A. irradians* (Du et al., 2017). For instance, in *P. yessoensis*, 395,646 SNPs were associated with the “Ivory” strain and 310,649 with the “Maple” strain (Ding et al., 2015). Among them, several SNPs impacted genes involved in metal transport, like the metalloreductase STEAP2 (Ding et al., 2015). In *A. irradians*, association analysis indicated that 126 SNPs were associated with carotenoid accumulation in the adductor muscle, like the cytochrome P450 family (Du et al., 2017). Whole genome sequencing of separate individuals remains costly for an association approach, especially for nonmodel species, and the number of sequenced individuals is often low. A way to use more individuals at a reduced cost is to perform reduced‐representation sequencing (like RAD‐seq). However, the small proportion of the genome that is sequenced makes it easy for polygenic associated variants to not be detected. Sequencing pools of individuals (Pool‐seq) represent another way to include many individuals while keeping the cost of sequencing down. It has been successful in identifying 17 SNPs linked to different color phenotypes in natural *Drosophila* populations (Bastide et al., 2013) and thus represents a cost‐effective technique to assess the association of variants to phenotypes by comparing allelic frequencies among those phenotypes. Moreover, this technique provides a great compromise between the sequencing costs (lower), the number of individuals sequenced (higher), and the coverage of the genome (whole genome).

The main objective of our study was to identify candidate SNPs associated with three inner shell color phenotypes using a total of 172 *P. margaritifera* individuals. For this, we used a pool‐seq strategy on three *P. margaritifera* inner shell colors of interest for pearl farming: red, yellow, and green phenotypes. The main questions were: (i) are there color‐associated SNPs to be identified in the red, green, and yellow phenotype, (ii) could these SNPs impact known pigmentation genes or pathways, and (iii) can we identify novel genes or pathways associated with color that were not identified in previous studies?

## 2 MATERIALS AND METHODS

### 2.1 Samples collection

**FIGURE 1:**
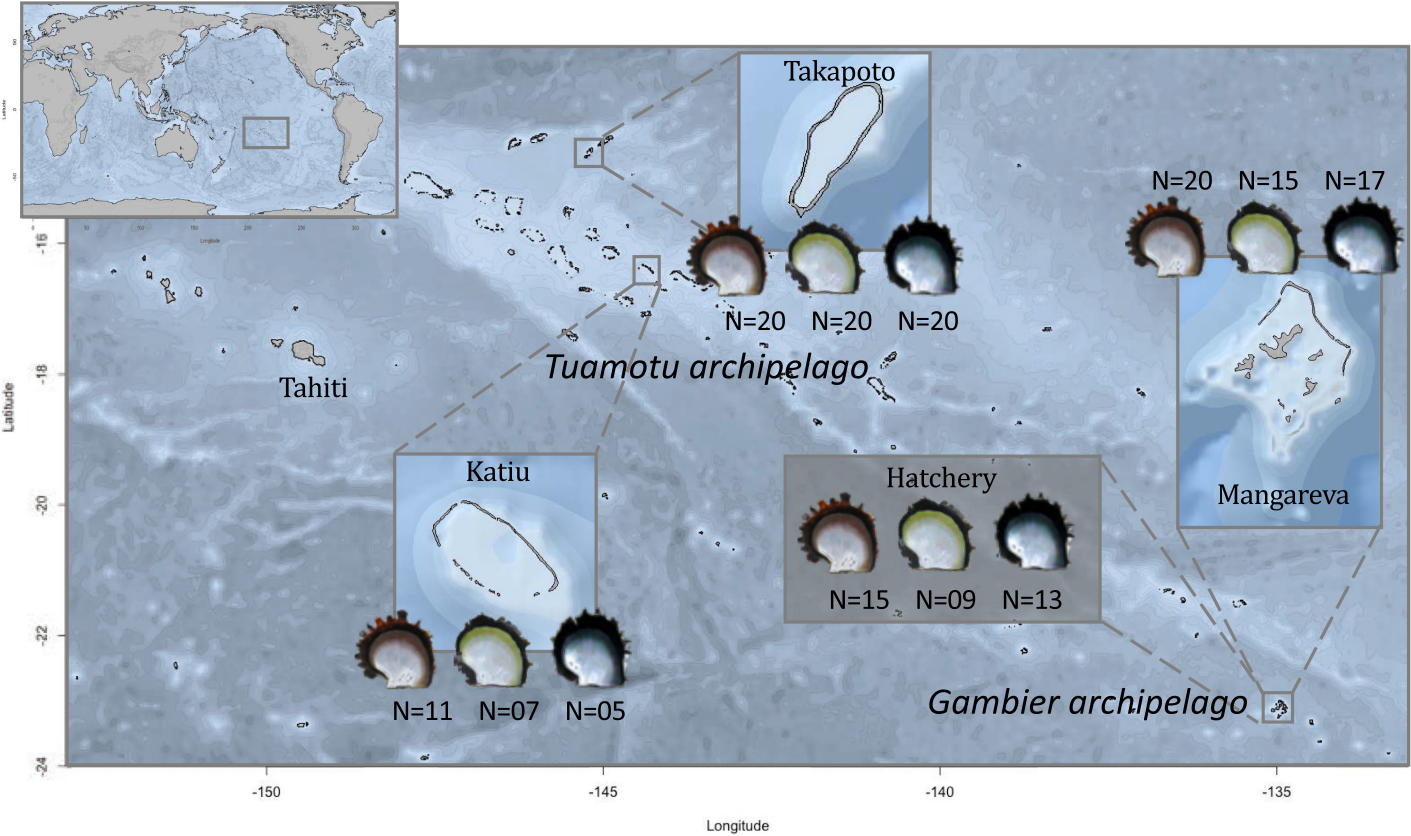
Map of French Polynesia showing the sampling sites and number of sequenced samples (*N*) per phenotype in this study: The hatchery individuals originated from the reproduction of wild individuals from the Gambier archipelago but were raised in the Ifremer lagoon concession in Tahiti.

In this study, we used three wild populations (Takapoto, Katiu, and Gambier) and one hatchery population selected for their strongly colored phenotypes and one hatchery‐produced population, and selected individuals from the three phenotypes in each population (see map in Figure 1). All individuals had a black mantle phenotype. The map was obtained with the R package *Marmap* V. 1.0.3 (Pante & Simon‐Bouhet, 2013, 2019) with the NOAA’s (National Oceanic and Atmospheric Administration of U.S.A.) bathymetry data. The wild individuals were collected from Takapoto (14°37′00.5″S 145°12′11.4″W) and Katiu (16°25′59.1″S 144°21′11.9″W) atolls in the Tuamotu archipelago and Mangareva (23°06′55.8″S 134°59′03.6″W) island in the Gambier archipelago.

For each location and each color phenotype, 50 individuals were selected by the visual appreciation of their color and phenotypically confirmed later by analyzing their inner shell color with *ImaginR* V 2.0 R package (Stenger, 2017; Stenger et al., 2019) (see Section 2.2), resulting in a total of 450 individuals for the wild group. An additional 20 samples per color phenotype were selected from a hatchery color breeding program (total *N* = 60). These F1 individuals originate from three multiparental families selected for their highly saturated color phenotypes (10 males and 10 females from each identified phenotype sampled from the Gambier archipelago contributed to the production of the progeny in the multiparental families through mass spawning). These F1 individuals were used to enhance the genetic signal of putative allelic variants linked with color phenotype, by reducing the genetic noise due to genetic diversity in the dataset. On the other hand, wild populations are used to avoid any family effect and false‐positive association due to a possibly reduced genetic diversity in F1. As such, a total of 510 samples were used for DNA extraction.

All individuals were sampled in 2017 and had a dorsoventral size between 8 and 12 cm for an estimated age of 2–3 years (age of maximum expression of their color phenotype; C.‐L. Ky, personal communication). For each individual, a 5 mm^3^ piece of mantle was sampled and stored in ethanol 96% at 4°C. After 48 h, ethanol was renewed. The shells of all individuals were cleaned and kept for inner shell color analysis.

### 2.2 Color typing and analysis

Shells of the sampled individuals were photographed with a Canon® PowerShot G9 placed in a Packshot Creator™ (V. 3.0.3.8), in order to prevent dark shadows and light reflection on the shell surface, as described in Stenger et al. (2019). Image processing and color qualification were realized in *ImaginR*, as described in Stenger et al. (2019). Using the HSV (Hue Saturation Value) color space, “ImaginR” characterizes inner shell color variations using a machine learning protocol with a training dataset. The training dataset allows for building a distribution of HSV values that corresponds to the different color phenotypes (giving the software pictures of what is considered red, yellow, or green phenotype in our species). The three HSV distributions of our species did not overlap, allowing for perfect identification. To phenotype a new individual, the picture of its shell is processed by the program, and its HSV values are extracted and compared with the distribution of the training dataset. to reassign shells (and pearls) to predefined phenotypic categories constructed from the training set. Any HSV combination falling outside of the profiles of the three phenotypes will be classified as “unknown,” while the rest of the individuals will be assigned to one of the three phenotypes. Individuals presenting both very high DNA quality and a validated affiliation to the red, yellow, or green phenotype categories in *ImaginR* were kept for sequencing. Pairwise *t*‐tests were conducted between phenotypes and sites for the HSV values, to test for any differences among geographical sites.

### 2.3 DNA extraction, QC, and pooling of samples

Samples were cleaned with DEPC before DNA extraction. Total DNA of 25 mg of mantle was extracted for each individual with a QIAamp DNA Mini Kit from QIAGEN® (Cat No./ID: 51306) following manufacturer recommendations, with an RNase A treatment step (see manual). An additional step was added after overnight digestion in order to remove mucopolysaccharides following the protocol in Sokolov (2000): 50 μl of a saturated KCl solution (34 g KCl/100 g H_2_O) was added to the digested samples, and the mixed solution was then centrifuged at 14,000 *g* for 15 min. DNA quality and quantity were assessed with a NanoDrop™ 1000 and agarose gels (500 ml of agarose 1.2%, TBE 1X, SYBR® Safe 3 μl [Invitrogen ‐ Cat No./ID: S33102], 50 volts). Only high‐quality DNA extraction was added to the pools. Pools were constructed using equimolar proportions of each individual of each phenotype and each sampling location. Figure 1 presents the number of sequenced individuals by pools according to their location and their phenotypes.

### 2.4 Sequencing approach

In total, 172 individuals were pooled according to their color and their population of origin, resulting in 12 pools (Figure 1). DNA libraries were prepared by Genome Québec (MPS Canada) facilities, using a TruSeq DNA PCR Free for Illumina Kit for DNA libraries/Shotgun PCR Free (800 ng DNA for library construction, Illumina TruSeq LT). Samples were sequenced on 12 lanes of an Illumina HiSeqX by the Genome Québec facilities (MPS Canada [one pool per lane]).

### 2.5 Read trimming, mapping, and SNP calling

Analyses were performed on the Ifremer Datarmor cluster. Raw read quality was assessed with FastQC (Andrews, 2010). Reads were cleaned, and adaptors were removed with Trimmomatic (Bolger et al., 2014) (V. 0.36—illuminaclip 2:30:10; leading 28; trailing 28; and minlen 40). BWA‐MEM V. 0.7.15 (Li & Durbin, 2009) was used to map reads against *P. margaritifera*'s genome assembly (Reisser et al., 2020). The 12 BAM files were filtered with SAMtools V. 1.4.1 (Li et al., 2009) to keep only uniquely mapped and properly paired reads with a mapping quality score higher than 5, and then sorted and indexed. PCR duplicates were removed using the Picard MarkDuplicates (V. 1.119), and reads were corrected with SplitNCigarReads from GATK (McCormick et al., 2015). BAM files were then indexed with SAMtools and checked with the flagstat tools.

Variant calling was performed with Freebayes V. 1.2.0 (Garrison & Marth, 2012). The pooled‐discrete and cnv‐map parameters allowed us to precise the number of individuals (*N* individuals times ploidy) used for each pool in order to adapt the SNP calling to the sample size. VCF Filter V.1.0.0 (Müller et al., 2017) was used to filter out SNPs with a depth below 20. We used VCFtools V. 0.1.14 (Danecek et al., 2011) to filter out SNP loci with a minor allele frequency below 1% (remove rare alleles) and with any missing genotyping. BCFtools V. 1.4.1 (Narasimhan et al., 2016) was used to keep only biallelic SNPs loci. 22,169,780 SNPs were obtained at this step. Complex events reported by Freebayes (biallelic block substitutions) were decomplexed with *vt* and the decompose_blocksub algorithm (Tan et al., 2015), thereby recovering 1848 additional SNPs.

### 2.6 Multivariate analysis

Pi diversity was calculated using VCFtools V. 0.1.14 (Danecek et al., 2011) with a window of 10,000 sites for each pool. A Kruskal–Wallis test and a pairwise Wilcoxon rank‐sum test were proceeded. For each pool, allelic frequencies (AF) from the VCF file were obtained by extracting the ratio of AD/DP for each of the SNPs.

To visualize relationships among samples, a principal component analysis was performed with the R packages *stats* V.3.6.1 (R Core Team) and *ggplot2* V.3.2.0 (Wickham, 2016) using AF data for each of the 12 pools.

### 2.7 Statistical models for association study

We used a combination of general linear model approaches to test for the presence of any significant differences in AF among color phenotypes while controlling for the geographical site of origin. Methodology for the combination of these statistical models was found in Hsu et al. (1996), Searle and Gruber (1971), Hothorn et al. (2008), and Crawley (2007).

For the GLM, the AF values were used as the response variable, while site and color values were used as explanatory variables.

$\text{for}\;\left(i\right)\;\mathrm{SNP}:$

$\mathrm{mod}\;\text{=}\;\mathrm{glm}\left(\mathrm{AF}∼\text{Color}+\text{Site}\right).$

Since there are two explanatory variables (color and site), the information was decomposed with another model because we were interested in comparing the levels of the two factors simultaneously. As such, a general linear hypothesis (GLHT) and multiple comparisons for parametric models (MCP) with ANOVA and post hoc Tuckey was implemented in a two‐step strategy, separating color and site testing.

The GLHT‐MCP was first used on the color results. The input model of the GLHT‐MCP was the result of the first GLM, and an ANOVA–Tukey test was performed for the MCP results. Results were stored in an R variable and the same process was used for the site variable and stored in another R object.

To identify SNPs related to both color and geography to remove them from the results (because we are here interested in the universal control of color), we performed an additional test that encompasses all the results: Simultaneous comparison of the levels of each factor was done using a new simple GLHT on the first GLM with the specification of the linear hypotheses of both GLHT‐MCP performed on color (K1) and onsite (K2):

$\text{glht}\left(\mathrm{mod},\text{linfct}=\text{rbind}\left[K1,K2\right]\right).$

A Bonferroni correction method (Hommel, 1988) was applied to all *p* values. All analyses previously cited were performed with the R packages *multcomp* V. 1.4–10 (Hothorn et al., 2019), *readr* V. 1.3.1 (Wickham, Hester, et al., 2022), *dplyr* V. 0.8.3 (Wickham, François, et al., 2022), *tidyr* (Wickham & Girlich, 2022), and *stringr* V. 1.4.0 (Wickham, 2019). Parallelization of the calculations was achieved with the R packages *foreach* V. 1.4.4 and *doParallel* V. 1.0.14 following Weston and Calaway (2019) recommendations. This model combination allowed us to obtain *p* values for all site comparisons and color phenotype comparisons. A SNP was deemed significantly associated with color if it had a Bonferroni corrected *p* value <1e10^−9^ in at least one pairwise color comparison and no geographical pairwise comparisons. In addition, a significantly associated SNP was deemed “color‐specific” if its association was found in all the pairwise comparisons involving a specific color (e.g., a green‐specific SNP will have a significant *p* value for both the green vs. yellow comparison, and for the green vs. red comparison).

The R package *circlize* (Gu et al., 2014) (V. 0.4.6) was used to create a chord diagram to visualize the color‐associated SNPs. Histograms for allelic frequency distributions were obtained with ggplot2 with 30 bins. We used pirate plots (concatenating raw data, descriptive statistics, and inferential statistics from the *yarrr* R package V.0.1.5; Phillips, 2017) to illustrate the differences between the 12 distributions. After checking data normality with a Shapiro test, a nonparametric Pairwise Wilcoxon test with a Bonferroni adjustment was performed with the R package *stats* to test any statistical differences between the distributions.

### 2.8 Functional analysis of associated SNPs

SnpEff and SnpSift V. 4.3 (Cingolani et al., 2012) were used to identify the potential impact of color‐associated SNPs on *P. margaritifera*’s gene sequence. Visualization of these results was obtained with the R packages *ggplot2* and *cowplot* V1 (Claus & Wilke, 2019). The annotated VCF files containing the color‐associated SNPs are available as File S01A–C in Appendix S1.

We then continued the analysis keeping only color‐associated SNPs with high, moderate, and modifier impacts: High category includes impacts like stop/start codon loss/gain, moderate category includes impacts like change in amino acid, and modifier category includes impacts like intergenic region, intron, or upstream/downstream variant (2000 bp around the gene, corresponding to the default parameter in SNPeff). GOATOOLS (Klopfenstein et al., 2018) was used to test for enrichment of GO terms in those SNPs, with the Fisher's exact test (significance at *p* value < 0.05). Significantly enriched GO categories were visualized in REVIGO (Supek et al., 2011) for the biological process, cellular components, and the molecular functions. The word clouds of the enriched GO terms was obtained with the OmicsBox software (biobam) using the top 40 most enriched GO terms (see File S02 in Appendix S1).

### 2.9 Amino acid changes and protein modeling

For all associated SNPs occurring in exonic regions of genes, complete exonic sequences were extracted using the *intersect* tool from BEDTOOLS (Quinlan & Hall, 2010). The corresponding exon sequence with the SNP location was translated into amino acid sequences with the Expasy web interface (https://www.expasy.org). The obtained amino acid sequences of these alternative proteins (bearing the associated variants) were compared with *P. margaritifera*'s protein sequences, and the corresponding frame in Expasy was selected. Protein modeling for the reference and alternative sequences were obtained for *P. margaritifera's* PBGD (porphobilinogen deaminase), shematrin, cytochrome P450 3A29, and Cadherin‐23 isoform X1 (see Stenger, Ky, Reisser, Duboisset, et al., 2021): (i) Sequence alignment were obtained with ClustalW for the *P. margaritifera* genome reference sequence and the alternative sequence carrying the amino acid modification and (if any) with other species referential sequences, (ii) I‐Tasser (https://zhanglab.ccmb.med.umich.edu/I‐TASSER/) was used for secondary structure prediction for both reference and alternative *P. margaritifera*'s amino acid sequences and for finding crystal structure (PDB) or the ligand binding site, (iii) superimposition of the Protein Database files was performed on the website tool SuperPose (http://superpose.wishartlab.com) with default parameters.

## 3 RESULTS

### 3.1 Color typing

Inner shell color analysis of the wild individuals revealed that 87.36%, 86.96%, and 98.3% of the sampled individuals were successfully assigned to the red, yellow, and green phenotypes, respectively. The remaining individuals had color patterns that did not allow proper phenotypic characterization (multiple colors on the shell, as described in Ky, Lo, et al., 2017) and were thus removed from further analysis (see Figure 1 for details on sampling design).

Within a given color phenotype, the average hue value obtained with *ImaginR* did not significantly differ among sites (red phenotype 0.032, 0.032, 0.031, 0.036; green phenotype: 0.490, 0.500, 0.490, 0.500; yellow phenotype: 0.220, 0.210, 0.210, 0.180 for Takapoto, Katiu, Gambier, and hatchery, respectively). The darkness parameter of the yellow pool for the hatchery population is significantly different from Gambier (*T*‐test *p* = 0.011) but not from Tuamotu.

### 3.2 Bioinformatics analysis

Between 875,890,480 and 978,499,888, raw sequences were obtained for the 12 pools (see Table 1 for lane‐specific results). After trimming, between 8.46% and 16.08% of sequences were removed and between 73.08% and 75.66% of sequences were properly mapped and paired to the reference genome. After filtering steps (see Section 2 for details) 22,171,628 SNPs were retained for statistical analysis.

**TABLE 1:** Number of sequences after the different bioinformatic steps for each of the 12 pools and the number of sequences that are mapped against the reference genome. The last row gives the mean of each column.

Color phenotype	Geographical site	Raw sequences	After trimming	Percent of deleted sequences	Number of properly paired against the genome	Percent of properly paired against the genome	Number of properly paired after filters	Percent of properly paired after filters
Red	Takapoto	933,451,922	804,525,582	16.02	652,910,874	81.15	491,651,124	75.30
Red	Katiu	919,931,270	825,237,588	11.47	675,882,634	81.90	506,143,676	74.89
Red	Gambier	977,173,146	900,905,220	8.46	746,316,126	82.84	545,397,822	73.08
Red	Hatchery	875,890,480	757,998,130	15.55	631,284,132	83.28	472,079,359	74.78
Yellow	Takapoto	938,782,098	821,910,616	14.22	671,154,850	81.66	507,808,553	75.66
Yellow	Katiu	943,199,888	866,874,884	8.80	709,770,466	81.88	522,722,833	73.65
Yellow	Gambier	978,499,586	884,295,558	10.65	729,092,800	82.45	540,454,292	74.13
Yellow	Hatchery	898,214,928	790,138,682	13.68	653,342,130	82.69	490,391,020	75.06
Green	Takapoto	911,432,464	785,142,178	16.08	632,289,464	80.53	472,054,295	74.66
Green	Katiu	898,068,018	785,537,094	14.32	649,464,162	82.68	475,609,233	73.23
Green	Gambier	949,968,516	867,379,084	9.52	713,228,138	82.23	530,586,280	74.39
Green	Hatchery	901,035,306	789,581,510	14.11	654,043,378	82.83	490,202,797	74.95
	Mean	927,137,301	823,293,843	12.74	676,564,929	82.18	503,758,440	74.48

### 3.3 Multivariate analysis and genetic diversity

**FIGURE 2:**
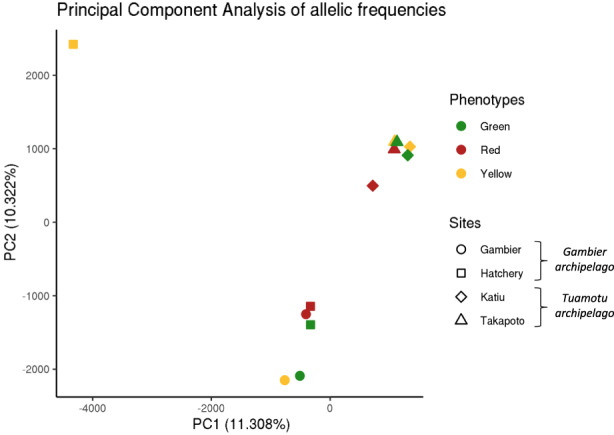
Principal component analysis (PCA) of the 12 pooled samples based on allelic frequencies (AF) at the 22,171,628 SNPs loci.

The Principal Component Analysis performed on allelic frequencies (AF) for the 12 pools preferentially groups the samples by geographical sites and not by color phenotypes (Figure 2): Takapoto and Katiu (the two atolls from the Tuamotu archipelago) are grouped together, while the Gambier and hatchery pools (the hatchery population originating from Gambier crossings) form another group. One exception to that is the yellow phenotype from the hatchery pool, which is differentiated from both the Tuamotu and the Gambier samples and represents an outlier.

Pi diversity does not statistically differ among the pools (see Files S03A,B in Appendix S1), or among the samples grouped according to geographical sites or color phenotypes.

### 3.4 Association study

**FIGURE 3:**
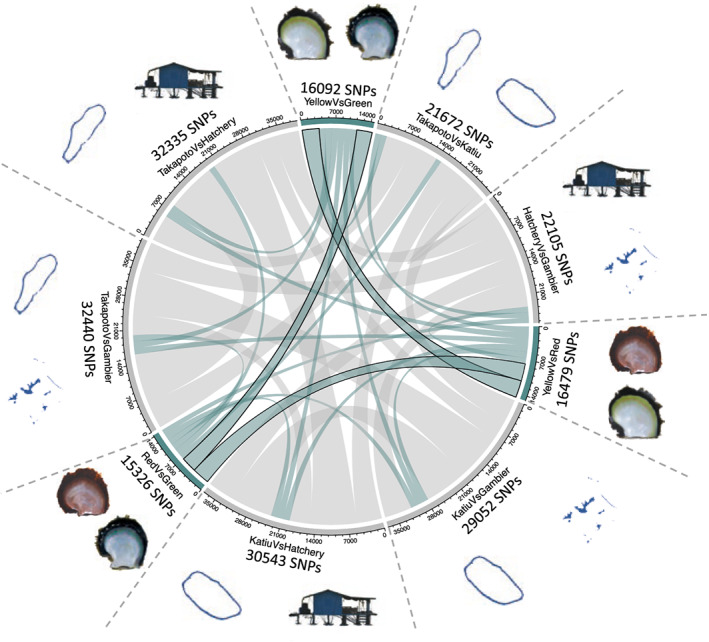
Chord diagram of significant SNPs (*p* value < 1e10^−9^) obtained across the geographical locations and color phenotypes. Gray chords represent the SNPs associated with geography. The color‐associated SNPs are indicated by green chords, while color‐only‐associated SNPs (SNPs that were not also geographically associated) are delimited by black borders.

We identified between 15,326 and 32,440 significantly associated SNPs depending on the color phenotype and geographical site considered (Figure 3). As expected, there were more SNPs found in association with the geographical origin (from 21,672 to 32,440 SNPs) than with color phenotype (from 15,326 to 16,479 SNPs). Of these, 3622 SNPs were specifically associated with the red phenotype, 3564 to the yellow, and 3240 to the green (10,426 SNPs in total). These color‐associated SNPs are delimited by a black border in the chords of the diagram in Figure 3.

**FIGURE 4:**
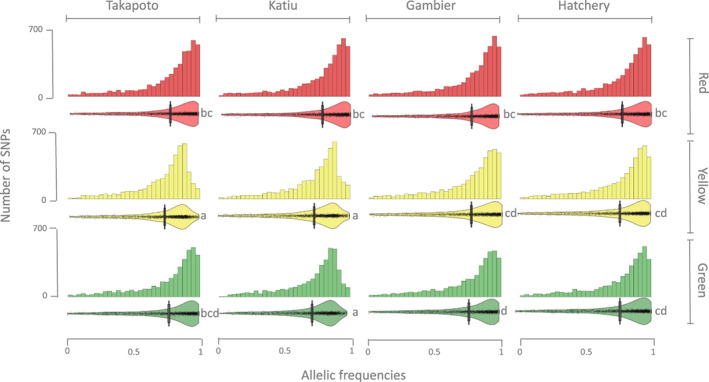
Allelic frequencies distributions of the color‐only‐associated SNPs according to their original pool. Histograms shows the AF distribution with 30 bins, while pirate plots (raw data, descriptive statistics, and inferential statistics plotted in same a graphics) show raw AF distribution data (points), the median (bar/line), the 95% HDI (high‐dimensional inference) (mustache), and the data density. Letters beside these plots correspond to the significance in the difference by the pairwise Wilcoxon test.

Allelic frequencies distributions of the color‐associated SNPs are shown in Figure 4. All distributions are skewed toward the alternative allele (higher homozygosity for the alternate allele), while the distribution for the 22,171,628 SNPs (associated and nonassociated) is skewed toward the reference allele (higher homozygosity for the reference allele). Pairwise Wilcoxon test showed that the AF distribution among red pools is more homogeneous than that among the yellow or among the green pools themselves. Moreover, the statistical differences of the Pairwise Wilcoxon test between AF distributions (Figure 4) did not correlate with the differences in hue, saturation, or darkness values between sites and phenotypes previously identified through the pairwise *t*‐test.

### 3.5 Functional analysis of color‐associated SNPs

**FIGURE 5:**
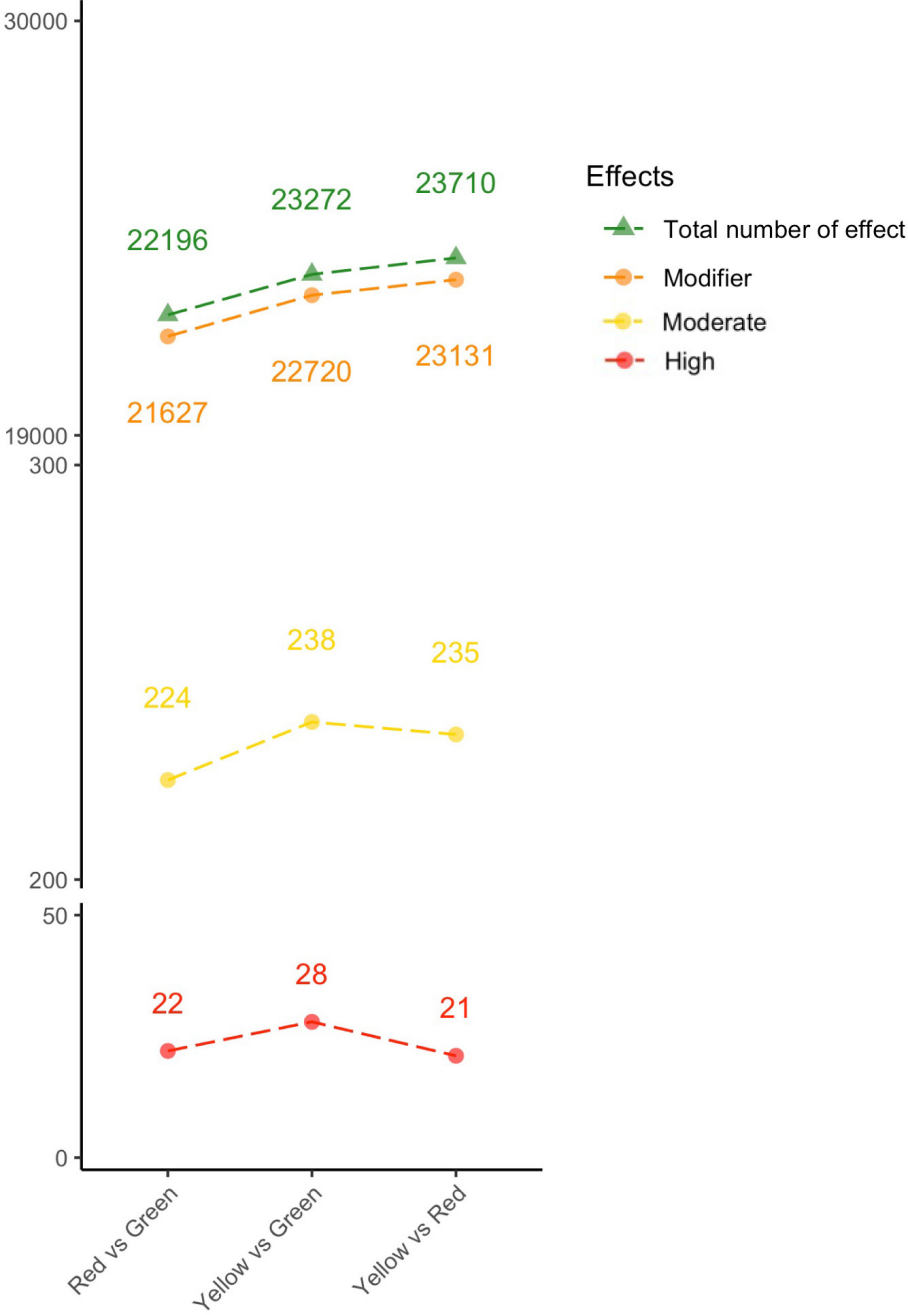
Number of SNPs impacts depending on pairwise color phenotype comparisons. The total number of effects contains all shown impact categories of the plot and low, missense, nonsense, and silent impacts.

Figure 5 shows the number of SNPs classified by their impact for each comparison. The modifier category contained the highest number of SNPs (97.61% of the total number of effects on average). The moderate and high categories only represented 1.05% and 0.09% of the total number of effects, respectively. Table 2 shows the most enriched GO terms according to GOATOOLS for every three phenotypes. The three color phenotypes share 12 enriched GO categories among which 3 were common to the red and green, 3 between red and yellow, and 4 between yellow and green. Interestingly, there were 31 color‐specific enriched GO terms: 11 GO terms were specifically enriched in the red phenotype, for example, “hydroxymethylbilane synthase activity” (GO:0004418), 11 GO terms were specifically enriched in the yellow phenotype, such as “calcium ion binding” (GO:0005509), and 9 GO terms were specifically enriched in the green phenotype like “heterocyclic compound binding” (GO:1901363) (Table 2).

**TABLE 2:** Enriched GO terms in color phenotypes according to GOATOOLS analyses, listing the terms found in the three phenotypes, those found in two phenotypes, and those that are phenotype‐specific.

Enriched GO terms in common for the three phenotypes
Protein binding (GO:0005515)
Binding (GO:0005488)
Catalytic activity (GO:0003824)
Ion binding (GO:0043167)
Anion binding (GO:0043168)
Small molecule binding (GO:0036094)
Nucleotide binding (GO:0000166)
Ribonucleotide binding (GO:0032553)
Purine nucleotide binding (GO:0017076)
Purine ribonucleoside triphosphate binding (GO:0035639)
Nucleoside phosphate binding (GO:1901265)
Enzyme binding (GO:0019899).
Enriched GO terms in common between red and green phenotype
Purine ribonucleotide binding (GO:0032550)
Identical protein binding (GO:0042802)
Transcription regulatory region sequence‐specific DNA binding (GO:0000976)
Enriched GO terms in common between red and yellow phenotype
Drug binding (GO:0008144)
Carbohydrate derivative binding (GO:0097367)
Catalytic activity(GO:0003824)
Enriched GO terms in common between green and yellow phenotype
Transporter activity (GO:0005215)
Transmembrane activity (GO:0022857)
Ion transmembrane transporter activity (GO:0015075)
Inorganic molecular entity transmembrane transporter activity (GO:0015318)
Enriched GO terms red‐specific
Adenyl nucleotide binding (GO:0030554)
Adenyl ribonucleotide binding (GO:0032559)
ATP binding (GO:0005524)
Hydroxymethylbilane synthase activity (GO:0004418)
Protein dimerization activity (GO:0046983)
Protein heterodimerization activity (GO:0046982)
DNA‐binding transcription factor activity (GO:0003700)
Enzyme regulator activity (GO:0030234)
Anion transmembrane transporter activity (GO:0008509)
Transcription regulator activity (GO:0140110)
Regulatory region nucleic acid binding (GO:0001067)
Enriched GO terms yellow‐specific
Calcium ion binding (GO:0005509)
G protein‐coupled receptor activity (GO:0004930)
Molecular transducer activity (GO:0060089)
Cation binding (GO:0043169)
Metal ion binding (GO:0046872)
Signaling receptor activity (GO:0038023)
Transmembrane signaling receptor activity (GO:0004888)
Transferase activity (GO:0016740)
Hydrolase activity (GO:0016787)
Phosphoric diester hydrolase activity (GO:0008081)
Hydrolase activity acting on ester bonds (GO:0016788)
Enriched GO terms green‐specific
Organic cyclic compound binding (GO:0097159)
Heterocyclic compound binding (GO:1901363)
Coenzyme binding (GO:0050662)
Cytoskeletal protein binding (GO:0008092)
Oxidoreductase activity (GO:0016491)
Sequence‐specific DNA binding (GO:0043565)
Double‐stranded DNA binding (GO:0003690)
Cofactor binding (GO:0018160)
RNA polymerase II regulatory region DNA binding (GO:0001012)

### 3.6 Pigmentation pathways associated with SNPs

Of the 10,426 color‐specific associated SNPs (see File S01A–C in Appendix S1), only 1.5% had GO categories related to pigment trafficking and biomineralization processes, like the encapsulation of molecules, vacuole trafficking pigments, or biomineralization. Here we focused on SNPs impacting genes directly involved in pigmentation (these SNPs can be found in Files S04 A–C in Appendix S1). A total of 54 different pigmentation‐related proteins encoded by 108 different transcripts were impacted by SNPs (Table 3). Red‐specific SNPs impacted 18 different genes encoding various pigment‐related proteins, like β, β ‐Carotene 15,15′‐dioxygenase gene, porphobilinogen deaminase, and cytochrome P450 2C8 (Table 3). Yellow‐specific SNPs impacted, 66 different pigment‐related genes, such as xanthine dehydrogenase/oxidase, cytochrome P450 3A11‐like, or many Copper/zinc superoxide dismutases (Table 3). Green‐specific SNPs impacted 27 pigment‐related genes, such as flavin reductase (NADPH), glutathione S‐transferase 1‐like, or three cytochrome P450 2C8‐like proteins (Table 3). Other genes were impacted by different SNPs in two color phenotypes: Cytochrome P450 4F8‐like and the visual pigment receptor peropsin‐like (RRH) genes were impacted by different SNPs identified in yellow and red phenotypes. Cytochrome P450 2C8‐like was also impacted by different SNPs in green and yellow phenotypes (Table 3).

**TABLE 3:** Name of pigment‐related proteins impacted by SNPs specific to the green, yellow, and green phenotype. For each protein, the information for one SNP is given as an example, with the name of this SNP (scaffold number and position of the SNP), the reference (R) and alternative (A) allele, their impact, and the number of transcripts bearing SNPs coding the same protein.

Gene name	SNPs number		SNP	R	A	Impact	Number of other similar genes
Red‐specific							
Beta,beta‐carotene 15,15′‐dioxygenase	1	Example for one SNP	scaffold605|size173110_50152	A	T	Upstream gene	0
Bifunctional purine biosynthesis protein PURH	1		scaffold8173|size41510_24501	T	G	Upstream gene	0
Cytochrome P450 26A1‐like	1		scaffold6003|size54905_33122	A	C	Intergenic	0
Cytochrome P450 2C8	4		scaffold417|size388363_270987	A	T	Intergenic	1
Cytochrome P450 3A29	1		scaffold2294|size169326_22277	C	T	missense_variant	0
Cytochrome P450 3A4	1		scaffold1969|size103307_48966	T	C	Intron	0
Cytochrome P450 4F8‐like	1		scaffold655|size199117_114782	C	A	Upstream gene	0
Porphobilinogen deaminase	14		scaffold2460|size144317_61211	G	A	Upstream gene	4 (see below)
Porphobilinogen deaminase	4		scaffold2460|size144317_128208	T	C	Intergenic	4 (see above & below)
Porphobilinogen deaminase	2		scaffold5737|size147992_39135	A	C	Downstream gene	4 (see above & below)
Porphobilinogen deaminase‐like	1		scaffold1000|size145372_49694	C	A	Intergenic	4 (see above & below)
Porphobilinogen deaminase‐like	3		scaffold2460|size144317_56369	T	C	Upstream gene	4 (see above)
Shematrin‐like protein 1	13		scaffold2460|size144317_86009	T	A	Upstream gene	0
Tyrosinase 2	1		scaffold1567|size137386_55806	G	A	Upstream gene	0
UDP‐glucuronosyltransferase 1–2	1		scaffold953|size134644_10904	T	A	Downstream gene	0
Versicolorin reductase‐like	1		scaffold4800|size171772_160414	C	T	Intergenic	0
Yellow‐specific							
Amorphous calcium carbonate‐binding protein 1	4	Example for one SNP	scaffold97|size234723_13738	T	A	Upstream gene	1
Cadherin‐23 isoform X1	9		scaffold5945|size55440_53306	C	G	Upstream gene	4
Copper/zinc superoxide dismutase	47		scaffold1412|size117757_38094	C	T	Upstream gene	37
Cytochrome b5 reductase 4‐like isoform X3	1		scaffold3263|size165871_73814	T	A	Intergenic	0
Cytochrome P450 2C8‐like	1		scaffold417|size388363_126041	T	G	Upstream gene	0
Cytochrome P450 3A11‐like isoform X1	2		scaffold9123|size36889_30428	G	A	Intron	1
Cytochrome P450 4F8‐like	1		scaffold5925|size155237_135045	C	T	Intergenic	0
Hypoxanthine‐guanine phosphoribosyltransferase	2		scaffold1749|size108746_82049	T	C	Intron	0
Laccase‐4‐like isoform X1	1		scaffold337|size296661_26903	T	C	Upstream gene	0
Laccase‐6	1		scaffold4044|size72409_7801	T	G	Upstream gene	0
Tyrosinase B4	2		scaffold2426|size145584_113288	C	T	Intergenic	1
Tyrosinase BPmax1	2		scaffold5775|size102228_21643	G	A	Intergenic	0
Tyrosinase‐like protein 1	4		scaffold1797|size279199_75418	A	G	Intergenic	1
Tyrosine‐protein kinase PR2 isoform X1	2		scaffold10826|size62665_1539	A	G	Downstream gene	0
Tyrosine‐protein kinase transmembrane receptor Ror	1		scaffold1018|size159565_123029	A	T	Intron	0
Tyrosine‐protein phosphatase nonreceptor type 11	1		scaffold5286|size98312_75622	T	A	Intron	0
Tyrosine‐protein phosphatase nonreceptor type 23	2		scaffold4726|size85232_2882	C	T	Intergenic	0
Visual pigment‐like receptor peropsin	1		scaffold1490|size115539_64221	T	A	Intergenic	0
Vitamin D(3) 25‐hydroxylase	1		scaffold5047|size126357_6322	C	A	Upstream gene	0
Xanthine dehydrogenase/oxidase	1		scaffold5976|size55229_32252	A	G	Downstream gene	0
Green‐specific							
Cadherin‐23	4	Example for one SNP	scaffold910|size252188_14555	T	C	Intron	3
Calcium‐binding protein CML8	1		scaffold88|size277366_192751	A	G	Intron	0
Calcium‐binding protein LPS1‐alpha‐like	1		scaffold14161|size18149_12593	A	T	Intron	0
Cytochrome P450 20A1	2		scaffold791|size153329_87835	T	C	Intron	0
Cytochrome P450 2C8‐like	4		scaffold49|size265340_38705	A	C	Intron	3
Cytochrome P450 4A25	1		scaffold1341|size119781_6168	C	T	Downstream gene	0
Cytochrome P450 4F12‐like	2		scaffold1676|size364098_330215	A	T	Intergenic	0
Ferritin 1	1		scaffold551|size158850_20422	A	C	Upstream gene	0
Ferrochelatase, mitochondrial	1		scaffold5249|size131904_104547	G	A	Intergenic	0
Flavin reductase (NADPH)	1		scaffold2158|size160354_118513	T	C	Intergenic	0
Glutathione S‐transferase 1‐like	1		scaffold1115|size353168_63863	A	T	Intron	0
Perlucin‐like protein	4		scaffold3547|size129194_105367	A	T	Downstream gene	2
Tyrosinase 1	2		scaffold454|size172198_160132	T	C	Intergenic	0
Tyrosinase B2.1	1		scaffold1980|size291657_59414	C	G	Intron	0
Urease subunit alpha‐like	2		scaffold101|size233448_106064	A	G	Intron	0
Visual pigment‐like receptor peropsin	1		scaffold2909|size388074_257210	T	A	Downstream gene	0

### 3.7 Amino acid changes and protein modeling

Among the 108 pigment‐related genes impacted by SNPs, only four had SNP impacting exonic sequences: a porphobilinogen deaminase (PBGD) sequence (scaffold2460|size144317.10), a shematrin sequence (scaffold2460|size144317.17), a cytochrome P450 3A29 (CYP3A29) (scaffold2294|size169326.2), and a Cadherin‐23 isoform X1 (scaffold5945|size55440.1). The SNP impacting the PBGD sequence induced a change from a 307 threonine into a 307 isoleucine; the SNP impacting the shematrin sequence induced a change from a 340 aspartic acid to a 340 asparagine; the SNP impacting the CYP3A29 induced a change from a 140 leucine to a 140 phenylalanine and the SNP impacting the Cadherin induced a change from a 119 threonine into a 119 alanine.

**FIGURE 6:**
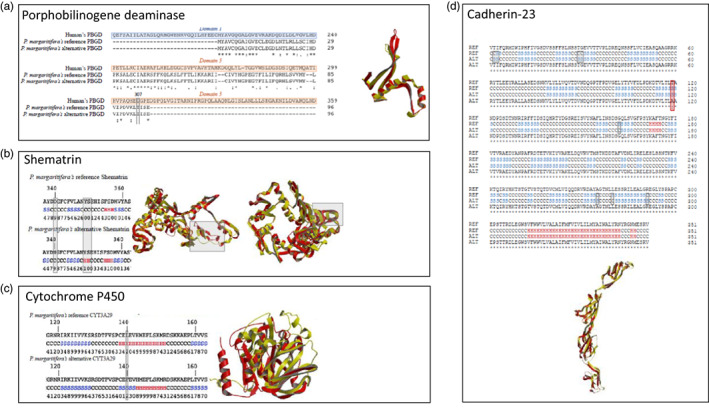
Protein modeling for the reference (genome sequence) and alternative pearl oyster sequence (carrying a SNP). (a) Modeling for the porphobilinogen deaminase (PBGD): ClustalW alignment of the reference and alternate sequence of *P. margaritifera* (gray box highlights the SNP position) along with the superimposition of the Protein Data Base files for reference (red) and alternative (yellow) sequences. (b) Modeling for the shematrin: ClustalW alignment of the reference and alternate sequence of *P. margaritifera* and predicted secondary structure (gray boxes highlight the SNP position (left) and the secondary structure modification (right)), along the superimposition of both reference (red) and alternative (yellow) shematrin Protein Databank file (gray boxes locate the differences on the superimposition). (c) Modeling for the CYT3A29: ClustalW alignment of the reference and alternate sequence of *P. margaritifera*, predicted secondary structure (gray box highlights both the SNP position and the secondary structure modification), along with the superimposition of both reference (red) and alternative (yellow) CYT3A29 Protein Databank file. (d) Modeling for the cadherin‐23: ClustalW alignment of the reference and alternate sequence of *P. margaritifera* and their corresponding secondary structure (gray boxes highlight the secondary structure modification, and the red box shows the SNP position), along the superimposition of both sequences reference (red) and alternative (yellow).

Alignment of the human reference PBGD (Song et al., 2009) sequence with the *P. margaritifera* genome's reference sequence and the alternative one revealed that both *P. margaritifera* sequences show a 31.58% similarity to the human PBGD (Figure 6a). The presence of the SNP reduces the similarity between the two *P. margaritifera* sequences at 98.96%. The secondary structure prediction by I‐Tasser showed that the AA substitution leads to the folding of a beta‐strand at the C‐terminal part of the alternative *P. margaritifera*'s PBGD sequence (see File S05A,B in Appendix S1). The superimposition between the reference and the alternative pearl oyster PBGD has a score of 488.8 (needle program; Figure 6a), indicating significant 3D conformation differences.

Since there is no shematrin reference protein in the Protein Data Bank, no match with shematrin protein was found with our de novo pearl oyster shematrin PDB. The comparison of the secondary structure of both reference and alternative *P. margaritifera*'s shematrin sequences shows that the change from 340 aspartic acid to 340 asparagine in the alternative sequence induces the folding of an alpha helix, from position 349 to 350 (Figure 6b). The protein modeling visualization indicates that the reference and alternative sequences are very close to each other at the tertiary structure level (Figure 6b, File S05C,D in Appendix S1). The superimposition needle score for both pearl oyster shematrins is relatively high, with a value of 1959.0.

For CYP3A29, the protein modeling of reference and alternative pearl oyster CYP3A29 highlight structural differences between them (File S05E,F in Appendix S1). While the reference sequence displays an alpha helix from the amino acid 138 to 151, the alternative sequence displays a beta‐strand from the amino acid 138 to 142 followed by an alpha helix from the amino acid 143 to 151 (Figure 6c). Despite the high needle score (1341.0) for the reference and alternative pearl oyster CYT3A29 sequences, the superimposition of their PDB is offset, due to the secondary structure changes linked to the SNP (Figure 6c).

Alignment of the *P. margaritifera*’s reference Cadherin‐23 isoform X1 sequence and the alternative indicated that both sequences are very similar (99.72%) while the alignment of the secondary structure shows a lower similarity (97.72%) (Figure 6d, File S05G,H in Appendix S1). The superimposition of both reference and alternative pearl oyster cadherin‐23 matches with a small needle score of 111.0 (Figure 6d), indicating a divergence in the conformation between the proteins.

## 4 DISCUSSION

The major quality trait of the French Polynesian pearls relies on the very diverse color range of the inner shell of *P. margaritifera* var. *cumingii*, and as such of its cultured pearls, a diversity that cannot be found in other *Pinctada* species (Ky et al., 2013; Ky, Le Pabic, et al., 2017). However, little is known about the putative genetic control of these colors in pearl oyster species (Lemer et al., 2015; Shinohara et al., 2018; Williams, 2016). Our aim was thus to identify SNPs associated with three different inner shell colors in *P. margaritifera* (red (Ky et al., 2015), yellow (Ky et al., 2015), and green (Ky, Le Pabic, et al., 2017)) to better understand the pathways involved in pigment production and the associated genetic mutations that impact them. While we identified SNPs impacting a set of genes that had been previously identified as differentially expressed among the different color phenotypes (thus highlighting a possible genetic control of their expression), we also uncover previously unknown pathways impacted by color‐specific SNPs, implicating novel candidate genes for the synthesis of pigment and expression of color phenotype in the Tahitian pearl oyster.

When considering both the geographical origin and the color of oysters, our results showed that geographical origin was the main driver of genetic differentiation among our samples. Indeed, samples were generally clustered by archipelago, except for the yellow individuals from the hatchery, which were outliers to all other samples. In wild populations, the yellow phenotype can present peculiarities regarding the positioning of the colored area of the inner shell: The colored area for the yellow individuals was indeed generally found at the bottom of the posterior section of the inner shell, while for the two other phenotypes, the colored spot was present as a large band in all posterior, connection, middle, and anterior sections of the inner shell (Ky et al., 2018). Moreover, results from *ImaginR* color qualification showed that the yellow hatchery individuals present a specific hue compared with all other yellow phenotypes while having a significantly different global darkness value than that of the Gambier individuals. While it suggests the existence of a peculiar pigmentation deposition for the yellow phenotype between hatchery's individuals and the others. It cannot be explained by a possible family effect or diversity effect, since allelic frequencies distribution (AFD) of the color‐associated SNPs indicated that the yellow hatchery AFD was similar to the other yellow AFD. In addition, levels of Pi diversity were found to be similar in all our samples, so that a possible inbreeding effect in the yellow hatchery sample is unlikely. Thus, the origin of the genetic variation and the outlier status of the yellow hatchery population cannot be identified at this stage but is to be noted.

### 4.1 Putative genetic control of the red phenotype

Our results demonstrated that the red phenotype might be controlled by a limited number of SNPs and might be less polygenic than the green and yellow phenotypes (Slatkin, 1978; Stahl et al., 2012). Indeed, the red phenotype had a very similar AF distribution in all the geographical sites, while the two other phenotypes showed more variation. This observation is also congruent with the phenotypic segregation observed in F1 experimental crosses between red individuals from previous studies (see Introduction).

The red‐specific SNPs showed functional enrichment for hydroxymethylbilane synthase activity (GO:0004418), a pathway containing the gene porphobilinogen deaminase (PBGD). This GO category was already identified as specific to the red color in a previous study (Stenger, Ky, Reisser, Duboisset, et al., 2021), in which PBGD was significantly downregulated in the red phenotype compared with the yellow and green phenotypes. This downregulation causes a form of acute intermittent porphyria (AIP) in mammals, linked with the accumulation of uroporphyrin I in cells, a well‐known red pigment that was previously identified in marine bivalve shells (Stenger, Ky, Reisser, Duboisset, et al., 2021). This kind of porphyria may here be the result of a nonfunctional PBGD protein due to a SNP (Balwani & Desnick, 2012; Meyer et al., 1972; Schneider‐Yin et al., 2002; Siersema et al., 1990; Song et al., 2009). Five different genes encoding porphobilinogen deaminases were found to be impacted by 91 SNPs in the red phenotype, though only one was located within an exon. This SNP created a beta‐strand at positions 92–93 of the protein and a 3D conformation change with the reference protein. However, we did not find any scientific literature on the cost of this change for the protein's functionality. The other SNPs were located in introns or upstream/downstream of the gene sequence, all of which are known to influence the expression levels of the neighboring genes (Shima et al., 2006; Tokuhiro et al., 2003). These SNPs could thus lead to a perturbation of the heme pathway, leading to an accumulation of the red pigment uroporphyrin I due to downregulation of the PBGD, in the red pearl oyster phenotype, as previously hypothesized (Stenger, Ky, Reisser, Duboisset, et al., 2021), and validation by experimental crosses, phenotyping, and genotyping will be necessary.

In addition to the previously identified heme pathway, our genome‐wide study highlighted a new pigmentation pathway not previously identified as involved in the red phenotype: the carotenoid pathway. Carotenoids are well‐known red, brown, and yellow pigments (Hornero‐Méndez et al., 2000; Lado et al., 2015; Tolmach & Graham, 1942). A SNP was located in the 5′ regions of the β, β ‐Carotene 15,15′‐dioxygenase gene (BCO1) and has a higher frequency in the red phenotype than others. BC01 is a key enzyme in beta‐carotene metabolism to vitamin A, and SNPs located in 5′ regions of genes involved in this pathway are known to impact the production of vitamin A (Lietz et al., 2012). Four other genes impacted by SNPs were found to intervene in carotenoids biosynthesis and in the correlated retinol metabolic pathway: three cytochromes P450 (CYP26A1, CYP2C8, and CYP3A29), impacted by 8 SNPs, and a UDP‐glucuronosyltransferase 1–2 (UGT), a gene previously described as differentially regulated in different color phenotypes in pearl oysters (Stenger, Ky, Reisser, Duboisset, et al., 2021). For the cytochrome P450 family, one SNP was located in an exonic region of CYP3A29. This SNP significantly changed the tertiary conformation of the protein by shortening an alpha helix and creating a new beta‐strand at the modified amino acid position, which suggests a possible impact on its functionality. SNPs affecting BCO1, CYP26A1, CYP2C8, CYP3A29, and UGT could affect concentrations of circulating carotenoids by modifying the degradation of β‐carotene molecules into all‐trans‐retinoic acids, leading to an accumulation of β‐carotene that would have to be excreted/degraded into pigmented carotenoids forms (Biesalski et al., 2007; Chichili et al., 2005; Hill & Johnson, 2012; Hornero‐Méndez et al., 2000; Lado et al., 2015; Lietz et al., 2012; Nadin & Murray, 1999; Naveed et al., 2018; Tolmach & Graham, 1942). Recently, retinol and retinoic genes have been confirmed to be associated with the shell color of *C. gigas* in the orange and black phenotype (Li et al., 2022).

The purine metabolism pathway also was impacted by a SNP upstream of the bifunctional purine biosynthesis protein (PurH ‐ EC:2.1.2.3 3.5.4.10 in the red phenotype). PurH controls the synthesis of FAICAR molecule (5′‐Phosphoribosyl‐5‐formamido‐4‐imidazolecarboxamide) and can also use PurH as a substrate to produce IMP (Inosine 5′‐monophosphate), the final molecule in the de novo purine nucleotide pathway (Beardsley et al., 1998). IMP molecule is the direct precursor to Guanosine 5′ triphosphate (GTP) (Ng et al., 2009) and was identified as differentially methylated and potentially involved in the coloration process in the pearl oyster *P. margaritifera* (Stenger, Ky, Reisser, Cosseau, et al., 2021). This specific GTP is involved in the synthesis of yellow pigments in the pterins pathways (xanthopterins and sepiapterins) and in pheomelanin or in eumelanin in the Raper–Mason pathway (Stenger, Ky, Reisser, Cosseau, et al., 2021; Stenger, Ky, Reisser, Duboisset, et al., 2021). Since IMP can be created in different ways, the pigment production in pterins and Raper–Mason pathway could be slightly reduced in the red phenotype. We also identified 13 SNPs related to the Shematrin‐like protein 1. The superimposition of the reference and alternative shematrin show differential conformation in the C‐terminal end of the protein suggesting potentially weaker functionality. Shematrins are Mollusca proteins expressed specifically in the edge region of the mantle (Marie et al., 2012; Yano et al., 2006). These proteins could take part in the calcification of the prismatic layer of the pearl oyster shell (Yano et al., 2006), and were overexpressed in black strains compared with the albino strains in *P. margaritifera* (Lemer et al., 2015).

In conclusion, individuals of the red phenotype have specific SNPs impacting the PBGD genes, which could affect gene expression and lead to the accumulation of the red pigment uroporphyrin I in the shell, as described in previous work (Stenger, Ky, Reisser, Duboisset, et al., 2021). They also have SNPs impacting pigments from the pterin pathway, a pathway previously identified in (Stenger, Ky, Reisser, Cosseau, et al., 2021; Stenger, Ky, Reisser, Duboisset, et al., 2021). However, the present results identified a new pathway that was not described before: Indeed, we found multiple SNPs impacting genes of the carotenoid pathways.

### 4.2 Putative genetic control of the yellow phenotype

In the yellow phenotype, we identified 18 impacted genes involved in the melanin pathway, such as laccases and tyrosinases. The melanin pathway uses DOPA and DOPA quinone as precursors to synthesize eumelanin and pheomelanin. DOPA quinone can be used as a substrate by glutathione S‐transferase, which binds glutathione (Sonthalia et al., 2016) to produce glutathionyldopa, leading to pheomelanin (yellow to red color) instead of eumelanin (brown to black color) (Stenger, Ky, Reisser, Duboisset, et al., 2021).

Several yellow‐specific SNPs impacted the cytochrome P450 family. We identified two SNPs in two introns of cytochrome P450 3A (CYP3A). CYP3A4 metabolizes the anthocyanidin and anthocyanin pigments (Srovnalova et al., 2014) and was downregulated in yellow shells of *Pinctada fucata martensii* when compared to the darker shells (Xu et al., 2019). Three upstream and one intronic SNPs were also identified in CYP4F8, a gene that is downregulated in patients with lentigo (small reddish pigmented spot on the skin that induces freckles) compared to patients with normal skin (Shin, 2012). An upstream yellow‐specific SNP was found in a gene coding for vitamin D(3) 25‐hydroxylase (also known as cytochrome P450 2R1 – CYP2R1 ‐ EC 1.14.14.24). CYP2R1 intervenes specifically in the steroid biosynthesis and controls the synthesis of calcidiol from vitamin D3. Calcidiol can become calcitriol in the next steps, which is the active form of vitamin D. There is a huge literature about the role of vitamin D3 and skin coloration (Jablonski & Chaplin, 2010). Indeed, the downregulation of this gene is found in light skin types compared with darker skin in humans (Liu et al., 2015). The putative implication of this gene to the yellow *P. margaritifera* phenotype is further supported by the previous transcriptomic and epigenetic analyses between yellow and green phenotypes in *P. margaritifera* (Stenger, Ky, Reisser, Cosseau, et al., 2021; Stenger, Ky, Reisser, Duboisset, et al., 2021), which identified differential expression and methylation of many enzymes involved in steroids and in vitamin D3 pathways.

The purine salvage pathway (Sculley et al., 1992) was also impacted by yellow‐specific SNPs. Two SNPs were found in the hypoxanthine‐guanine phosphoribosyltransferase gene (HGPRT) in the yellow phenotype. HGPRT plays a central role in the generation of purine nucleotides, and its deficiency leads to the Lesch–Nyhan syndrome characterized by the overproduction of sand‐like crystals of uric acid (Sculley et al., 1992). Also, an upstream SNP impacting xanthine dehydrogenase/oxidase (XDH) was identified in the yellow phenotype. Xanthine dehydrogenase and related genes code for molybdenum‐containing hydroxylases that catalyze purine and pteridine reactions (Brondino et al., 2006). This family of genes contributes to red, orange, and yellow pigmentation in many animals (Pimsler et al., 2017; Shamim et al., 2014; Watt, 1972). This gene encodes an enzyme that oxidizes the yellow pteridine xanthopterin compound to the colorless pteridine leucopterin compound in *Colias* butterflies (Watt, 1972).

Other classically described genes involved in biomineralization processes were impacted by yellow‐specific SNPs. Three upstream and one downstream SNPs were found in the vicinity of two genes encoding for an amorphous calcium carbonate binding protein 1 (ACCBP). This gene is involved in the morphology of nacre lamellae in the shell of *Pinctada* species (Ma et al., 2007; Shi et al., 2013). Indeed, this gene could inhibit the growth of undesired aragonite crystal faces and cease the nucleation and growth of calcite (Ma et al., 2007). These could modify the morphology of nacre lamellae (Ma et al., 2007), and such changes could modify the color by physical mechanisms (Rousseau & Rollion‐Bard, 2012; Stenger, Ky, Reisser, Cosseau, et al., 2021), further studies on this gene could be interesting.

Finally, 38 different genes encoding putative copper/zinc superoxide dismutase (Cu/Zn SOD) were impacted by 47 yellow‐specific SNPs. Pigmentation pathways usually generate highly reactive molecules (porphyrins for example) that can be oxidized and have the potential to create reactive oxygen species (ROS), deleterious to the cell. Cu/Zn SOD are enzymes that catalyze the dismutation of the superoxide radical into either ordinary molecular oxygen or hydrogen peroxide. For example, Cu/Zn SOD expression is incredibly high in patients with vitiligo because of the melanogenesis pathway perturbation (Wacewicz et al., 2018).

In conclusion, the yellow individuals have SNPs impacting laccases, tyrosinases, HGPRT, and XDH, which could be the source of the differential expression identified in these genes previously (Stenger, Ky, Reisser, Duboisset, et al., 2021). In addition, our results identified new genes (CYP4F8, CYP3A4, and CYP2R1) in these pathways.

### 4.3 Putative genetic control of the green phenotype

A previous transcriptomic study (Stenger, Ky, Reisser, Duboisset, et al., 2021) found that the GST enzyme (see yellow phenotype above) which occurs in the melanin pathway is downregulated in the green phenotype compared with the yellow phenotype. GST uses dopaquinone to produce specifically pheomelanin, a yellow pigment. In the green phenotype, we found a green‐specific SNP in GST, which could explain the downregulation of this enzyme detected in previous studies (Stenger, Ky, Reisser, Duboisset, et al., 2021), leading to the preferential production of the black eumelanin pigment preferentially to the yellow pheomelanin pigment. Recently, the same hypothesis has been raised for the black *Pinctada fucata martensii* color line (Adzigbli et al., 2020). Moreover, it was proven recently that the regulation of expression of different tyrosinase genes (essential enzymes for melanism process) plays a vital role in the shell color of the bivalve *Ruditapes philippinarum* (Jiang et al., 2020).

One green‐specific upstream SNP impacted the ferritin 1 gene. This enzyme (EC:1.16.3.2) stores iron in a soluble, nontoxic, readily available form and could be used in the heme biosynthesis pathway (Theil et al., 2012; Wang et al., 2011). Also, one green‐specific SNP impacted ferrochelatase (FECH). FECH intervenes in the latest steps of the heme biosynthesis, to produce heme from protoporphyrin IX. Also belonging to the heme pathway, flavin reductase NADPH (BLVRB gene ‐ EC:1.3.1.24) was impacted by a green‐specific SNP. This enzyme converts biliverdin (green pigment) into bilirubin (yellow pigment) (Cunningham et al., 2000). The SNP impacting BLVRB could affect the synthesis of the protein, leading to green biliverdin accumulation in the green phenotype, as previously hypothesized (Stenger, Ky, Reisser, Duboisset, et al., 2021).

Thirteen SNPs impacting nine different cadherin‐23 genes (Figure 6b) were identified in our dataset, among which four SNPs impacted four green‐specific transcripts. Although cadherin‐23 could not be directly linked to any green pigment, this protein is involved in several pigmentation processes in other organisms. Cadherin‐23 overexpression has been associated with the “purple zigzag” phenotype in the clam *Meretrix*, with an upregulation in this phenotype compared with the white phenotype (Yue et al., 2015).

Classic biomineralization genes were also identified as possibly impacted by SNPs. Four green‐specific SNPs were identified in up‐ and downstream regions of three perlucin‐like genes. Perlucin is involved in the nucleation and subsequent growth of calcium carbonate crystals (Blank et al., 2003). This protein allows the connection between the chitin and aragonite layers (Sun et al., 2015). Recently, this protein was recognized to be linked to the pigmentation of the shell of *Ruditapes philippinarum* (Nie et al., 2020). Also, multiple SNPs were identified in calcium‐binding protein cml8 and calcium‐binding protein LPS1‐alpha‐like, which are potential calcium sensors found only in the green phenotype.

In conclusion, the green individuals have SNPs impacting the previously identified melanin pathway and the heme pathway. However, cadherins are new genes that were not previously described in color‐associated genes in *P. margaritifera*. These genes are impacted by several SNPs for the green phenotype and should be investigated in further studies.

## 5 CONCLUSION

The goal of the study was to look for genetic variants associated with three different color phenotypes of the Tahitian pearl oyster: green, yellow, and red. First, we confirmed that the genomic control of color in *P. margaritifera* is polygenic, with SNPs impacting multiple genes involved in several pigmentation pathways, sometimes shared among the different colors. Moreover, we noted the fact that the red phenotype was likely less polygenic than yellow and green phenotypes, since the allelic frequency variation of the associated SNPs among the four populations studied was not as strong as for the two other phenotypes. Of the 108 pigment‐related SNPs we identified, four fell into exonic sequences, while many others were located in upstream or downstream regions, in intergenic regions, or in introns. The four exonic SNPs impacted the 3D conformation of the proteins they modified, but their actual effect on gene expression and the resulting phenotype will have to be confirmed by further experimental studies, as is the case of upstream and downstream SNPs. Associated SNPs located in intergenic regions cannot be overlooked either, as they could impact LincRNAs and control color phenotypes, like for *C. gigas*.

Our whole genome pooled‐sequencing approach revealed that many genes impacted by color‐specific SNPs had been identified as candidate genes in the transcriptomic analysis of the same color phenotypes. However, our study highlighted complementary pathways not identified previously, and which are known to be involved in color expression in other Mollusca and animals, such as the carotenoids pathway for the red phenotype (BCO1). The presence of these pigments in the shell of the pearl oysters will have to be confirmed through biochemistry since the RAMAN spectroscopy analysis used in previous studies on the shells of the Tahitian pearl oysters is not effective at identifying carotenoids. Also, new candidate genes were identified for the yellow phenotype (CYP4F8, CYP3A4, and CYP2R1) and for the green phenotype (cadherin‐23). Finally, we identified genes that are not directly involved in pigment synthesis but that are known to intervene in the control of potential cell damage due to the reactive nature of many pigments (oxidative stress and ROS degradation) like the Cu/Zn SOD genes.

The actual functional role of the candidate SNPs in pigmentation pathways must be validated. Currently, it is not possible to cultivate mantle cell lineages of *P. margaritifera* in the lab, to provide a way to use crispR techniques and observe the color of nacre precipitation of these cells. RNAi techniques were tested but are very tedious to use on such a species, and very stressful for the animal. Currently, our only perspective is to use these SNPs and genotypes as many individuals as possible to validate the pattern of segregation we see here on a larger scale. A SNP Chip has been developed and includes all the SNPs identified in this study, to validate the association of these SNPs to color morphs. The chip is currently being tested in breeding programs with pearl farmers. Our results open new doors for the implementation of future breeding programs focused on individual genomic selection for specific color production in pearl oysters, and improve the footprint of perliculture on Polynesian lagoon by reducing the production volume while increasing the quality of the pearls.

## CONFLICT OF INTEREST

The authors have no conflicts of interest to disclose.

## Supporting information


Appendix S1

## Data Availability

Raw reads for this manuscript are openly available at the Sextant Database https://sextant.ifremer.fr/record/e0a8f7bc‐423d‐4043‐b997‐d839a083f01b/ (Reisser, 2018). The whole pipeline and code scripts can be found on GitHub (PLStenger/Pearl_Oyster_Colour_Population_Genomics/00_scripts).
