# Supplementary material for: Identifying genes associated with genetic control of color polymorphism in the pearl oyster Pinctada margaritifera var. cumingii (Linnaeus 1758) using a comparative whole genome pool‐sequencing approach
Source: Evol Appl. 2022 Sep 16;16(2):408–27. doi: 10.1111/eva.13464 (PMC9923487; doi:10.1111/eva.13464)

SUPPLEMENTARY FILES

File S01 A, B and C: Annotated VCF files of all color associated SNPs obtained through SNPEff.

File S2: Enrichment analysis of all color-associated SNPs with high, moderate and modifier impact – actual colors corresponding to the color phenotype: bottom for red, left for green and right for yellow.

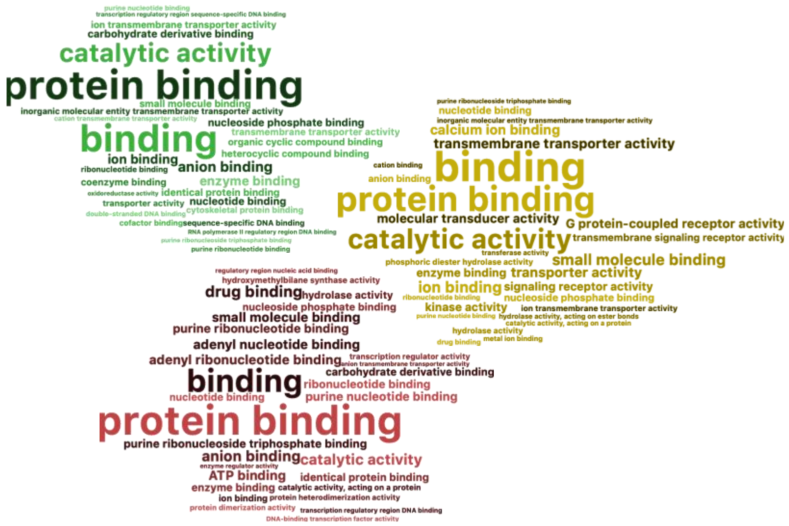

File S3:

A: Box plot of Pi diversity values by pool.

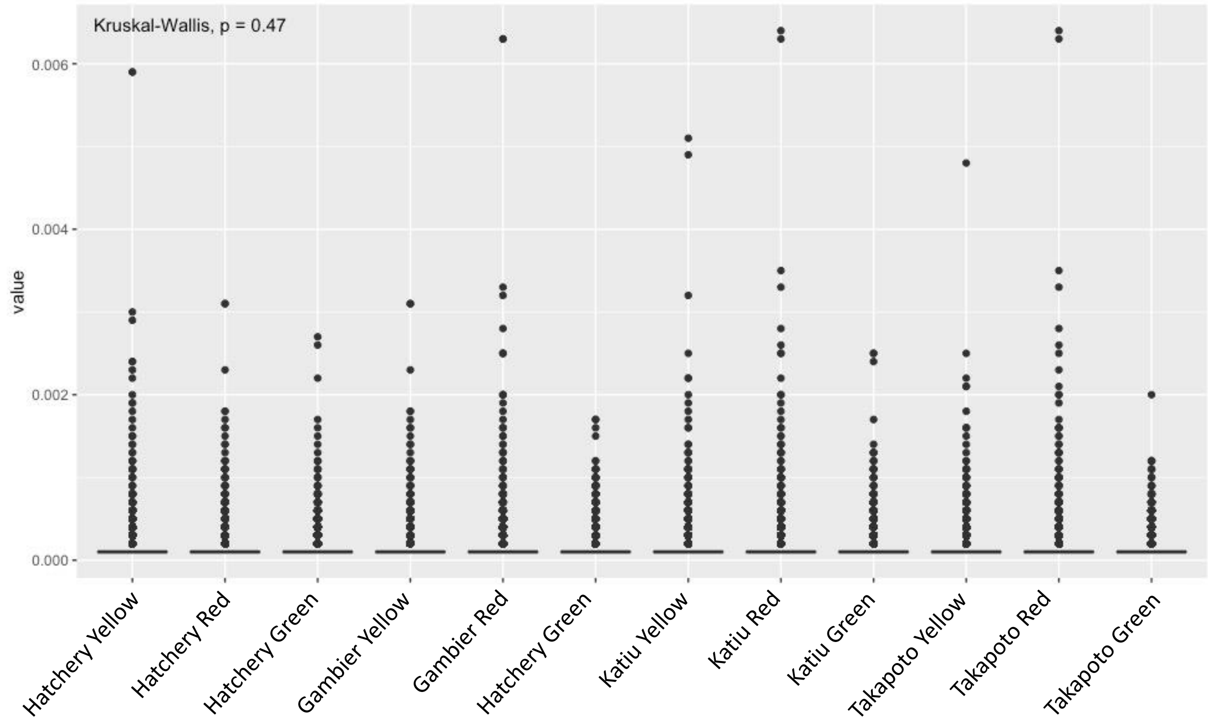

B: Matrix of a pairwise Wilcoxon rank sum test. Legend: HY (Hatchery Yellow), HR (Hatchery Red), HG (Hatchery Green), GY (Gambier Yellow), GR (Gambier Red), GG (Gambier Green), KY (Katiu Yellow), KR (Katiu Red), KG (Katiu Green), TY (Takapoto Yellow), TR (Takapoto Red), TG (Takapoto Green)

	**HY**	**HR**	**HG**	**GY**	**GR**	**GG**	**KY**	**KR**	**KG**	**TY**	**TR**
**HR**	1	-	-	-	-	-	-	-	-	-	-
**HG**	0.91	0.91	-	-	-	-	-	-	-	-	-
**GY**	1	1	0.91	-	-	-	-	-	-	-	-
**GR**	1	1	0.91	1	-	-	-	-	-	-	-
**GG**	0.91	0.91	0.98	0.91	0.91	-	-	-	-	-	-
**KY**	0.87	0.87	0.83	0.87	0.87	0.83	-	-	-	-	-
**KR**	0.91	0.91	0.91	0.91	0.91	0.91	0.91	-	-	-	-
**KG**	0.91	0.91	0.91	0.91	0.91	0.98	0.8	0.91	-	-	-
**TY**	0.92	0.91	1	0.91	0.92	0.92	0.83	0.91	0.91	-	-
**TR**	0.91	0.91	0.91	0.91	0.91	0.91	0.91	1	0.91	0.91	-
**TG**	0.91	0.91	0.91	0.91	0.91	0.91	0.47	0.83	0.91	0.91	0.83

File S4:

A - List of all red specific SNPs, from left to right: with the transcripts code; the proteins that are encoding by genes related to pigmentation impacted by the SNPs; the SNP place corresponding to the number of base (after the underscore) in the scaffold code; reference allele: alternative allele; impact of the SNP; and allelic frequencies for red, yellow and green phenotype.

transcripts	Gene name	SNP position	R	A	Impact	Red's AF	Yellow's AF	Green's AF
scaffold605|size173110.14	**Beta,beta-carotene 15,15'-dioxygenase**	scaffold605|size173110_50152	A	T	Upstream gene	0.515715817	0.509004081	0.484623714
scaffold8173|size41510.12	**Bifunctional purine biosynthesis protein PURH**	scaffold8173|size41510_24501	T	G	Upstream gene	0.952082147	0.92797624	0.853512908
scaffold6003|size54905.10	**Cytochrome P450 26A1-like**	scaffold6003|size54905_33122	A	C	Intergenic	0.708563225	0.751351468	0.812912089
scaffold417|size388363.34	**Cytochrome P450 2C8**	scaffold417|size388363_270987	A	T	intergenic_region	0.969551485	0.900845824	0.823202804
scaffold417|size388363.34	**Cytochrome P450 2C8**	scaffold417|size388363_359355	G	T	intergenic_region	0.9649712	0.933456183	0.925194938
scaffold4234|size70448.7	**Cytochrome P450 2C8**	scaffold4234|size70448_28221	C	T	upstream_gene_variant	0.695819849	0.739572216	0.817266452
scaffold3353|size151707.13	**Cytochrome P450 2C8**	scaffold3353|size151707_73590	A	T	intron_variant	0.736388464	0.722691234	0.594777798
scaffold2294|size169326.2	**Cytochrome P450 3A29**	scaffold2294|size169326_22277	C	T	missense_variant	0.898544452	0.931637807	0.815374396
scaffold1969|size103307.16	**Cytochrome P450 3A4**	scaffold1969|size103307_48966	T	C	Intron	0.859897047	0.835853166	0.889425918
scaffold655|size199117.18	**Cytochrome P450 4F8-like**	scaffold655|size199117_114782	C	A	Upstream gene	0.90864899	0.841163952	0.811255693
scaffold2460|size144317.18	**porphobilinogen deaminase**	scaffold2460|size144317_130621	A	G	intergenic_region	0.828496277	0.71108361	0.518735272
scaffold2460|size144317.18	**porphobilinogen deaminase**	scaffold2460|size144317_127821	A	G	intergenic_region	0.964370267	0.866413342	0.656325175
scaffold2460|size144317.18	**porphobilinogen deaminase**	scaffold2460|size144317_107033	T	G	intron_variant	0.665903799	0.583019936	0.47948978
scaffold2460|size144317.18	**porphobilinogen deaminase**	scaffold2460|size144317_107148	T	A	intron_variant	0.700719915	0.55800332	0.422491024
scaffold2460|size144317.18	**porphobilinogen deaminase**	scaffold2460|size144317_106662	C	T	intron_variant	0.666528787	0.597987144	0.444500422
scaffold2460|size144317.18	**porphobilinogen deaminase**	scaffold2460|size144317_111879	T	C	intron_variant	0.842650282	0.74895711	0.579734848
scaffold2460|size144317.18	**porphobilinogen deaminase**	scaffold2460|size144317_104040	T	G	intron_variant	0.906324618	0.783969505	0.612682063
scaffold2460|size144317.18	**porphobilinogen deaminase**	scaffold2460|size144317_113739	C	T	intron_variant	0.857869778	0.678095861	0.498014845
scaffold2460|size144317.18	**porphobilinogen deaminase**	scaffold2460|size144317_116741	C	T	upstream_gene_variant	0.31729625	0.389865536	0.558645328
scaffold2460|size144317.18	**porphobilinogen deaminase**	scaffold2460|size144317_100892	T	C	intron_variant	0.891666667	0.756257796	0.542574586
scaffold2460|size144317.18	**porphobilinogen deaminase**	scaffold2460|size144317_119689	A	G	upstream_gene_variant	0.896573542	0.783480933	0.613256095
scaffold2460|size144317.18	**porphobilinogen deaminase**	scaffold2460|size144317_112462	T	A	intron_variant	0.964691527	0.912632539	0.825923728
scaffold2460|size144317.18	**porphobilinogen deaminase**	scaffold2460|size144317_122017	A	G	intergenic_region	0.91012863	0.756422675	0.549519317
scaffold2460|size144317.18	**porphobilinogen deaminase**	scaffold2460|size144317_122150	G	T	intergenic_region	0.916733835	0.737264279	0.534101688
scaffold2460|size144317.18	**porphobilinogen deaminase**	scaffold2460|size144317_124355	G	A	intergenic_region	0.957396215	0.806595816	0.556655844
scaffold2460|size144317.18	**porphobilinogen deaminase**	scaffold2460|size144317_121774	G	C	intergenic_region	0.947950646	0.804084492	0.576817043
scaffold2460|size144317.18	**porphobilinogen deaminase**	scaffold2460|size144317_123137	T	G	intergenic_region	0.804626267	0.586279246	0.449776917
scaffold2460|size144317.18	**porphobilinogen deaminase**	scaffold2460|size144317_126279	A	G	intergenic_region	0.964442356	0.842533515	0.552757631
scaffold2460|size144317.18	**porphobilinogen deaminase**	scaffold2460|size144317_121759	A	T	intergenic_region	0.915761005	0.788619596	0.562122386
scaffold2460|size144317.18	**porphobilinogen deaminase**	scaffold2460|size144317_122869	A	T	intergenic_region	0.984394652	0.884359557	0.805719816
scaffold2460|size144317.18	**porphobilinogen deaminase**	scaffold2460|size144317_121746	A	T	intergenic_region	0.891050505	0.752344321	0.547415527
scaffold2460|size144317.18	**porphobilinogen deaminase**	scaffold2460|size144317_124468	C	A	intergenic_region	0.985685232	0.962953617	0.90502236
scaffold2460|size144317.18	**porphobilinogen deaminase**	scaffold2460|size144317_128208	T	C	intergenic_region	0.943561984	0.760625308	0.560228902
scaffold2460|size144317.18	**porphobilinogen deaminase**	scaffold2460|size144317_128185	A	G	intergenic_region	0.985429128	0.918620935	0.837672315
scaffold2460|size144317.18	**porphobilinogen deaminase**	scaffold2460|size144317_124550	G	A	intergenic_region	0.701986039	0.504498871	0.393742903
scaffold2460|size144317.18	**porphobilinogen deaminase**	scaffold2460|size144317_123772	T	A	intergenic_region	0.945785398	0.795710513	0.584338768
scaffold2460|size144317.18	**porphobilinogen deaminase**	scaffold2460|size144317_121985	A	G	intergenic_region	0.925352096	0.778086831	0.568765436
scaffold2460|size144317.18	**porphobilinogen deaminase**	scaffold2460|size144317_121822	A	T	intergenic_region	0.962972596	0.814359642	0.577639917
scaffold2460|size144317.18	**porphobilinogen deaminase**	scaffold2460|size144317_123216	A	T	intergenic_region	0.898595669	0.729846599	0.508126284
scaffold2460|size144317.18	**porphobilinogen deaminase**	scaffold2460|size144317_126338	T	C	intergenic_region	0.662421209	0.522709285	0.378684718
scaffold2460|size144317.18	**porphobilinogen deaminase**	scaffold2460|size144317_122908	G	A	intergenic_region	0.984678271	0.886806238	0.783206633
scaffold2460|size144317.18	**porphobilinogen deaminase**	scaffold2460|size144317_126540	G	A	intergenic_region	0.952428511	0.829941777	0.5693004
scaffold2460|size144317.18	**porphobilinogen deaminase**	scaffold2460|size144317_127498	T	A	intergenic_region	0.954684449	0.854040404	0.656436531
scaffold2460|size144317.18	**porphobilinogen deaminase**	scaffold2460|size144317_127334	G	T	intergenic_region	0.936801671	0.802443484	0.580567227
scaffold2460|size144317.18	**porphobilinogen deaminase**	scaffold2460|size144317_128552	A	C	intergenic_region	0.954579533	0.767631209	0.572462259
scaffold2460|size144317.18	**porphobilinogen deaminase**	scaffold2460|size144317_137696	G	A	intergenic_region	0.914337682	0.82155796	0.534197249
scaffold2460|size144317.18	**porphobilinogen deaminase**	scaffold2460|size144317_133554	C	A	intergenic_region	0.961924264	0.800211312	0.520893142
scaffold2460|size144317.18	**porphobilinogen deaminase**	scaffold2460|size144317_129755	G	A	intergenic_region	0.984824511	0.835174142	0.655804647
scaffold2460|size144317.18	**porphobilinogen deaminase**	scaffold2460|size144317_130159	A	T	intergenic_region	0.863986847	0.801534235	0.630855451
scaffold2460|size144317.18	**porphobilinogen deaminase**	scaffold2460|size144317_138489	G	T	intergenic_region	0.954599455	0.867370466	0.740896144
scaffold2460|size144317.18	**porphobilinogen deaminase**	scaffold2460|size144317_138484	T	C	intergenic_region	0.957600393	0.865104403	0.750117283
scaffold2460|size144317.18	**porphobilinogen deaminase**	scaffold2460|size144317_136001	T	A	intergenic_region	0.98751938	0.943909539	0.768852161
scaffold2460|size144317.18	**porphobilinogen deaminase**	scaffold2460|size144317_140782	T	C	intergenic_region	0.909830586	0.952312734	0.955488678
scaffold2460|size144317.18	**porphobilinogen deaminase**	scaffold2460|size144317_140942	G	A	intergenic_region	0.944625038	0.897870275	0.704351157
scaffold2460|size144317.18	**porphobilinogen deaminase**	scaffold2460|size144317_138391	C	T	intergenic_region	0.95385089	0.871794872	0.732637253
scaffold2460|size144317.18	**porphobilinogen deaminase**	scaffold2460|size144317_141495	C	T	intergenic_region	0.981827201	0.879357452	0.740940695
scaffold2460|size144317.18	**porphobilinogen deaminase**	scaffold2460|size144317_140776	T	C	intergenic_region	0.912393556	0.952485768	0.959038388
scaffold2460|size144317.18	**porphobilinogen deaminase**	scaffold2460|size144317_138208	C	T	intergenic_region	0.947437442	0.886201173	0.695264776
scaffold2460|size144317.18	**porphobilinogen deaminase**	scaffold2460|size144317_138828	T	C	intergenic_region	0.97053901	0.916774015	0.762742365
scaffold2460|size144317.18	**porphobilinogen deaminase**	scaffold2460|size144317_138902	A	G	intergenic_region	0.978492137	0.929313064	0.762269349
scaffold2460|size144317.18	**porphobilinogen deaminase**	scaffold2460|size144317_141184	C	A	intergenic_region	0.884573413	0.755815644	0.418243876
scaffold2460|size144317.18	**porphobilinogen deaminase**	scaffold2460|size144317_143102	A	C	intergenic_region	0.822060277	0.61527692	0.347017045
scaffold2460|size144317.18	**porphobilinogen deaminase**	scaffold2460|size144317_138868	A	G	intergenic_region	0.930386406	0.890933517	0.72861607
scaffold2460|size144317.18	**porphobilinogen deaminase**	scaffold2460|size144317_138383	A	G	intergenic_region	0.960714286	0.875744048	0.731063134
scaffold2460|size144317.18	**porphobilinogen deaminase**	scaffold2460|size144317_138396	G	T	intergenic_region	0.958122037	0.871428571	0.737155022
scaffold2460|size144317.18	**porphobilinogen deaminase**	scaffold2460|size144317_138974	A	G	intergenic_region	0.910337376	0.824505069	0.498773367
scaffold2460|size144317.18	**porphobilinogen deaminase**	scaffold2460|size144317_141958	G	T	intergenic_region	0.713869163	0.63582343	0.276612337
scaffold2460|size144317.18	**porphobilinogen deaminase**	scaffold2460|size144317_138639	G	T	intergenic_region	0.955442955	0.933051155	0.737635928
scaffold2460|size144317.18	**porphobilinogen deaminase**	scaffold2460|size144317_140864	A	T	intergenic_region	0.963915298	0.880784103	0.764850816
scaffold2460|size144317.18	**porphobilinogen deaminase**	scaffold2460|size144317_141468	A	C	intergenic_region	0.973639456	0.901566185	0.748913935
scaffold2460|size144317.18	**porphobilinogen deaminase**	scaffold2460|size144317_140816	T	C	intergenic_region	0.903081568	0.943751575	0.957636662
scaffold2460|size144317.18	**porphobilinogen deaminase**	scaffold2460|size144317_136302	G	T	intergenic_region	0.96432785	0.821428571	0.565551411
scaffold2460|size144317.18	**porphobilinogen deaminase**	scaffold2460|size144317_141298	A	T	intergenic_region	0.931163004	0.893004349	0.738821086
scaffold2460|size144317.18	**porphobilinogen deaminase**	scaffold2460|size144317_141482	T	A	intergenic_region	0.974188312	0.88938406	0.744135915
scaffold2460|size144317.18	**porphobilinogen deaminase**	scaffold2460|size144317_143161	G	T	intergenic_region	0.790453964	0.6125	0.282024325
scaffold2460|size144317.18	**porphobilinogen deaminase**	scaffold2460|size144317_136367	G	A	intergenic_region	0.974725275	0.834783208	0.550227039
scaffold2460|size144317.18	**porphobilinogen deaminase**	scaffold2460|size144317_141978	C	G	intergenic_region	0.700897436	0.592152894	0.290750518
scaffold2460|size144317.12	**porphobilinogen deaminase**	scaffold2460|size144317_60430	A	T	upstream_gene_variant	0.946500247	0.864542129	0.719184464
scaffold2460|size144317.12	**porphobilinogen deaminase**	scaffold2460|size144317_61312	C	T	upstream_gene_variant	0.93698882	0.828636929	0.668843401
scaffold2460|size144317.12	**porphobilinogen deaminase**	scaffold2460|size144317_59863	T	C	upstream_gene_variant	0.614753175	0.521662278	0.447971389
scaffold2460|size144317.12	**porphobilinogen deaminase**	scaffold2460|size144317_60477	A	G	upstream_gene_variant	0.953589053	0.860411473	0.715812867
scaffold2460|size144317.12	**porphobilinogen deaminase**	scaffold2460|size144317_62416	T	A	upstream_gene_variant	0.930114796	0.811417526	0.650907236
scaffold2460|size144317.12	**porphobilinogen deaminase**	scaffold2460|size144317_61305	C	T	upstream_gene_variant	0.930515216	0.819547256	0.676493156
scaffold2460|size144317.12	**porphobilinogen deaminase**	scaffold2460|size144317_63095	C	T	upstream_gene_variant	0.724529752	0.671141008	0.519202067
scaffold2460|size144317.12	**porphobilinogen deaminase**	scaffold2460|size144317_60471	T	C	upstream_gene_variant	0.940600328	0.866781791	0.710242554
scaffold2460|size144317.12	**porphobilinogen deaminase**	scaffold2460|size144317_61211	G	A	upstream_gene_variant	0.939839481	0.839995557	0.72941013
scaffold2460|size144317.12	**porphobilinogen deaminase**	scaffold2460|size144317_77776	G	A	intergenic_region	0.953098056	0.877291322	0.81556545
scaffold2460|size144317.12	**porphobilinogen deaminase**	scaffold2460|size144317_78746	G	T	intergenic_region	0.872097254	0.735651959	0.57557313
scaffold2460|size144317.12	**porphobilinogen deaminase**	scaffold2460|size144317_77147	C	T	intergenic_region	0.96574487	0.906226902	0.842519403
scaffold2460|size144317.12	**porphobilinogen deaminase**	scaffold2460|size144317_78649	T	G	intergenic_region	0.907095394	0.822537383	0.603257908
scaffold2460|size144317.12	**porphobilinogen deaminase**	scaffold2460|size144317_80093	T	A	intergenic_region	0.710518603	0.566431938	0.452406306
scaffold2460|size144317.18	**porphobilinogen deaminase**	scaffold2460|size144317_96901	C	T	intron_variant	0.886764091	0.807256415	0.646697154
scaffold2460|size144317.18	**porphobilinogen deaminase**	scaffold2460|size144317_97253	T	C	intron_variant	0.949924874	0.889820432	0.76341461
scaffold2460|size144317.18	**porphobilinogen deaminase**	scaffold2460|size144317_97023	A	G	intron_variant	0.924812245	0.8176626	0.646319407
scaffold2460|size144317.18	**porphobilinogen deaminase**	scaffold2460|size144317_97007	A	G	intron_variant	0.903663103	0.773599631	0.582490995
scaffold5737|size147992.15	**porphobilinogen deaminase**	scaffold5737|size147992_39135	A	C	downstream_gene_variant	0.717871832	0.624614441	0.471578712
scaffold5737|size147992.15	**porphobilinogen deaminase**	scaffold5737|size147992_39149	C	T	downstream_gene_variant	0.572685648	0.554948524	0.378707627
scaffold1000|size145372.7	**porphobilinogen deaminase-like**	scaffold1000|size145372_49694	C	A	intergenic_region	1	0.920668917	0.874370561
scaffold2460|size144317.10	**porphobilinogen deaminase-like**	scaffold2460|size144317_56350	A	G	upstream_gene_variant	0.923632917	0.82441949	0.671445638
scaffold2460|size144317.10	**porphobilinogen deaminase-like**	scaffold2460|size144317_52782	G	A	missense_variant	0.994444444	0.966062099	0.929897807
scaffold2460|size144317.10	**porphobilinogen deaminase-like**	scaffold2460|size144317_56369	T	C	upstream_gene_variant	0.922997902	0.824768839	0.677987221
scaffold2460|size144317.17	**Shematrin-like protein 1**	scaffold2460|size144317_85435	C	G	upstream_gene_variant	0.931240064	0.827928554	0.645783564
scaffold2460|size144317.17	**Shematrin-like protein 1**	scaffold2460|size144317_91830	T	C	downstream_gene_variant	0.727460474	0.501406403	0.313924837
scaffold2460|size144317.17	**Shematrin-like protein 1**	scaffold2460|size144317_92107	T	C	downstream_gene_variant	0.725283833	0.562705475	0.306811916
scaffold2460|size144317.17	**Shematrin-like protein 1**	scaffold2460|size144317_91879	A	G	downstream_gene_variant	0.727643285	0.552521186	0.333938701
scaffold2460|size144317.17	**Shematrin-like protein 1**	scaffold2460|size144317_84999	G	T	upstream_gene_variant	0.901565306	0.787465722	0.69299024
scaffold2460|size144317.17	**Shematrin-like protein 1**	scaffold2460|size144317_87800	C	A	upstream_gene_variant	0.90104516	0.73322528	0.520221563
scaffold2460|size144317.17	**Shematrin-like protein 1**	scaffold2460|size144317_87607	A	T	upstream_gene_variant	0.870395659	0.717818151	0.523477861
scaffold2460|size144317.17	**Shematrin-like protein 1**	scaffold2460|size144317_86009	T	A	upstream_gene_variant	0.933894231	0.806059564	0.697992802
scaffold2460|size144317.17	**Shematrin-like protein 1**	scaffold2460|size144317_85266	T	C	upstream_gene_variant	0.167130842	0.323286611	0.549759279
scaffold2460|size144317.17	**Shematrin-like protein 1**	scaffold2460|size144317_87367	T	A	upstream_gene_variant	0.823581787	0.690936325	0.480345851
scaffold2460|size144317.17	**Shematrin-like protein 1**	scaffold2460|size144317_92636	G	T	downstream_gene_variant	0.810062241	0.524615385	0.387722816
scaffold2460|size144317.17	**Shematrin-like protein 1**	scaffold2460|size144317_92525	A	G	downstream_gene_variant	0.816037132	0.642592593	0.428223057
scaffold2460|size144317.17	**Shematrin-like protein 1**	scaffold2460|size144317_91213	G	A	missense_variant	0.755464481	0.639567016	0.416393727
scaffold1567|size137386.11	**Tyrosinase 2**	scaffold1567|size137386_55806	G	A	Upstream gene	0.98139166	0.966537468	0.931356845
scaffold953|size134644.1	**UDP-glucuronosyltransferase 1-2**	scaffold953|size134644_10904	T	A	Downstream gene	0.923824122	0.93604907	0.944753577
scaffold4800|size171772.26	**Versicolorin reductase-like**	scaffold4800|size171772_160414	C	T	Intergenic	0.886998886	0.860606209	0.818379234

63

4

B - List of all yellow specific SNPs, from left to right: with the transcripts code; the proteins that are encoding by genes related to pigmentation impacted by the SNPs; the SNP place corresponding to the number of base (after the underscore) in the scaffold code; reference allele: alternative allele; impact of the SNP; and allelic frequencies for red, yellow and green phenotypes.

<table>
<tr><th>transcripts</th><th>Gene name</th><th>SNP position</th><th>R</th><th>A</th><th>impact</th><th>Red's AF</th><th>Yellow's AF</th><th>Green's AF</th></tr>
<tr><td>scaffold1219|size149844.2</td><td>amorphous calcium carbonate binding protein 1</td><td>scaffold1219|size149844_11628</td><td>T</td><td>G</td><td>upstream_gene_variant</td><td>0.925084502</td><td>0.911592087</td><td>0.962188279</td></tr>
<tr><td>scaffold1219|size149844.2</td><td>amorphous calcium carbonate binding protein 1</td><td>scaffold1219|size149844_11660</td><td>A</td><td>T</td><td>upstream_gene_variant</td><td>0.637810449</td><td>0.69471917</td><td>0.799332873</td></tr>
<tr><td>scaffold1219|size149844.2</td><td>amorphous calcium carbonate binding protein 1</td><td>scaffold1219|size149844_7885</td><td>T</td><td>G</td><td>downstream_gene_variant</td><td>0.692837846</td><td>0.655041066</td><td>0.718970047</td></tr>
<tr><td>scaffold97|size234723.4</td><td>amorphous calcium carbonate binding protein 1</td><td>scaffold97|size234723_13738</td><td>T</td><td>A</td><td>upstream_gene_variant</td><td>0.224785692</td><td>0.137438155</td><td>0.277067131</td></tr>
<tr><td>scaffold5945|size55440.1</td><td>cadherin-23 isoform X1</td><td>scaffold5945|size55440_53306</td><td>C</td><td>G</td><td>upstream_gene_variant</td><td>0.876784634</td><td>0.845343003</td><td>0.946941629</td></tr>
<tr><td>scaffold5945|size55440.1</td><td>cadherin-23 isoform X1</td><td>scaffold5945|size55440_49639</td><td>C</td><td>A</td><td>intron_variant</td><td>0.755842297</td><td>0.760117913</td><td>0.776755209</td></tr>
<tr><td>scaffold5945|size55440.1</td><td>cadherin-23 isoform X1</td><td>scaffold5945|size55440_48952</td><td>T</td><td>C</td><td>missense_variant</td><td>0.652283534</td><td>0.618162393</td><td>0.733237548</td></tr>
<tr><td>scaffold5945|size55440.1</td><td>cadherin-23 isoform X1</td><td>scaffold5945|size55440_49073</td><td>T</td><td>A</td><td>intron_variant</td><td>0.873202614</td><td>0.831136468</td><td>0.914658224</td></tr>
<tr><td>scaffold7897|size43079.5</td><td>cadherin-23 isoform X1</td><td>scaffold7897|size43079_25046</td><td>C</td><td>A</td><td>intron_variant</td><td>0.367920331</td><td>0.406697665</td><td>0.348014031</td></tr>
<tr><td>scaffold2505|size92724.2</td><td>cadherin-23 isoform X2</td><td>scaffold2505|size92724_60662</td><td>G</td><td>T</td><td>intron_variant</td><td>0.933719291</td><td>0.934110171</td><td>0.960124105</td></tr>
<tr><td>scaffold234|size272526.38</td><td>cadherin-23-like isoform X3</td><td>scaffold234|size272526_207359</td><td>C</td><td>T</td><td>intergenic_region</td><td>0.91686437</td><td>0.901875</td><td>0.869123872</td></tr>
<tr><td>scaffold2505|size92724.1</td><td>cadherin-23-like isoform X3</td><td>scaffold2505|size92724_18810</td><td>T</td><td>C</td><td>intron_variant</td><td>0.86537074</td><td>0.865843672</td><td>0.90107426</td></tr>
<tr><td>scaffold2505|size92724.1</td><td>cadherin-23-like isoform X3</td><td>scaffold2505|size92724_29819</td><td>A</td><td>G</td><td>intron_variant</td><td>0.896043358</td><td>0.892719733</td><td>0.912491856</td></tr>
<tr><td>scaffold562|size200826.1</td><td>copper/zinc superoxide dismutase</td><td>scaffold562|size200826_43820</td><td>G</td><td>A</td><td>intergenic_region</td><td>0.595933805</td><td>0.605798082</td><td>0.647571709</td></tr>
<tr><td>scaffold109|size389597.41</td><td>copper/zinc superoxide dismutase</td><td>scaffold109|size389597_223108</td><td>A</td><td>G</td><td>downstream_gene_variant</td><td>0.899460837</td><td>0.887365591</td><td>0.957576697</td></tr>
<tr><td>scaffold1092|size179208.14</td><td>copper/zinc superoxide dismutase</td><td>scaffold1092|size179208_121855</td><td>T</td><td>A</td><td>upstream_gene_variant</td><td>0.871486974</td><td>0.778905593</td><td>0.848507304</td></tr>
<tr><td>scaffold1092|size179208.10</td><td>copper/zinc superoxide dismutase</td><td>scaffold1092|size179208_89636</td><td>A</td><td>T</td><td>upstream_gene_variant</td><td>0.849744467</td><td>0.881638399</td><td>0.914382412</td></tr>
<tr><td>scaffold11014|size28661.2</td><td>copper/zinc superoxide dismutase</td><td>scaffold11014|size28661_2549</td><td>C</td><td>A</td><td>downstream_gene_variant</td><td>0.921393449</td><td>0.940526911</td><td>0.974760527</td></tr>
<tr><td>scaffold11014|size28661.2</td><td>copper/zinc superoxide dismutase</td><td>scaffold11014|size28661_2991</td><td>G</td><td>T</td><td>downstream_gene_variant</td><td>0.940681445</td><td>0.935659364</td><td>0.997368421</td></tr>
<tr><td>scaffold112|size675390.132</td><td>copper/zinc superoxide dismutase</td><td>scaffold112|size675390_512926</td><td>A</td><td>G</td><td>intergenic_region</td><td>0.844551182</td><td>0.809749212</td><td>0.797894725</td></tr>
<tr><td>scaffold112|size675390.132</td><td>copper/zinc superoxide dismutase</td><td>scaffold112|size675390_510586</td><td>A</td><td>G</td><td>intergenic_region</td><td>0.853862476</td><td>0.847902204</td><td>0.785847777</td></tr>
<tr><td>scaffold1183|size219875.2</td><td>copper/zinc superoxide dismutase</td><td>scaffold1183|size219875_24681</td><td>A</td><td>G</td><td>upstream_gene_variant</td><td>0.851810894</td><td>0.81789658</td><td>0.875464</td></tr>
<tr><td>scaffold12|size329199.6</td><td>copper/zinc superoxide dismutase</td><td>scaffold12|size329199_22276</td><td>C</td><td>T</td><td>upstream_gene_variant</td><td>0.783948928</td><td>0.793279368</td><td>0.924104344</td></tr>
<tr><td>scaffold1320|size225772.17</td><td>copper/zinc superoxide dismutase</td><td>scaffold1320|size225772_59142</td><td>G</td><td>T</td><td>upstream_gene_variant</td><td>0.937065972</td><td>0.943277367</td><td>0.92148927</td></tr>
<tr><td>scaffold1335|size119901.13</td><td>copper/zinc superoxide dismutase</td><td>scaffold1335|size119901_82315</td><td>A</td><td>T</td><td>intron_variant</td><td>0.222696102</td><td>0.163904526</td><td>0.240279752</td></tr>
<tr><td>scaffold1412|size117757.9</td><td>copper/zinc superoxide dismutase</td><td>scaffold1412|size117757_38094</td><td>C</td><td>T</td><td>upstream_gene_variant</td><td>0.327205662</td><td>0.382240027</td><td>0.253706423</td></tr>
<tr><td>scaffold1440|size256764.20</td><td>copper/zinc superoxide dismutase</td><td>scaffold1440|size256764_174679</td><td>G</td><td>T</td><td>upstream_gene_variant</td><td>0.929672108</td><td>0.933864469</td><td>0.908771556</td></tr>
<tr><td>scaffold1440|size256764.20</td><td>copper/zinc superoxide dismutase</td><td>scaffold1440|size256764_175328</td><td>C</td><td>T</td><td>upstream_gene_variant</td><td>0.806374384</td><td>0.80403291</td><td>0.84868088</td></tr>
<tr><td>scaffold1460|size244210.17</td><td>copper/zinc superoxide dismutase</td><td>scaffold1460|size244210_88734</td><td>C</td><td>G</td><td>upstream_gene_variant</td><td>0.82466041</td><td>0.897521802</td><td>0.895020522</td></tr>
<tr><td>scaffold1498|size160682.4</td><td>copper/zinc superoxide dismutase</td><td>scaffold1498|size160682_53036</td><td>T</td><td>C</td><td>intron_variant</td><td>0.806381844</td><td>0.823703836</td><td>0.89688258</td></tr>
<tr><td>scaffold1520|size200124.32</td><td>copper/zinc superoxide dismutase</td><td>scaffold1520|size200124_111755</td><td>A</td><td>T</td><td>upstream_gene_variant</td><td>0.880139191</td><td>0.881022192</td><td>0.939095606</td></tr>
<tr><td>scaffold1574|size113249.4</td><td>copper/zinc superoxide dismutase</td><td>scaffold1574|size113249_51961</td><td>T</td><td>C</td><td>intron_variant</td><td>0.872314458</td><td>0.868005479</td><td>0.942884199</td></tr>
<tr><td>scaffold1890|size140805.17</td><td>copper/zinc superoxide dismutase</td><td>scaffold1890|size140805_92739</td><td>A</td><td>C</td><td>upstream_gene_variant</td><td>0.789841644</td><td>0.726411819</td><td>0.693028283</td></tr>
<tr><td>scaffold1903|size152924.21</td><td>copper/zinc superoxide dismutase</td><td>scaffold1903|size152924_69697</td><td>T</td><td>C</td><td>intergenic_region</td><td>0.328205611</td><td>0.350407864</td><td>0.321598295</td></tr>
<tr><td>scaffold1928|size104068.10</td><td>copper/zinc superoxide dismutase</td><td>scaffold1928|size104068_42051</td><td>C</td><td>G</td><td>upstream_gene_variant</td><td>0.893775297</td><td>0.916398416</td><td>0.874064774</td></tr>
<tr><td>scaffold2390|size226504.12</td><td>copper/zinc superoxide dismutase</td><td>scaffold2390|size226504_57764</td><td>G</td><td>A</td><td>upstream_gene_variant</td><td>0.770950144</td><td>0.741096693</td><td>0.933031945</td></tr>
<tr><td>scaffold2390|size226504.12</td><td>copper/zinc superoxide dismutase</td><td>scaffold2390|size226504_60328</td><td>T</td><td>A</td><td>downstream_gene_variant</td><td>0.442775744</td><td>0.473117974</td><td>0.618026007</td></tr>
<tr><td>scaffold2456|size156049.1</td><td>copper/zinc superoxide dismutase</td><td>scaffold2456|size156049_24185</td><td>C</td><td>T</td><td>intergenic_region</td><td>0.579226447</td><td>0.521491013</td><td>0.487870629</td></tr>
<tr><td>scaffold316|size347727.2</td><td>copper/zinc superoxide dismutase</td><td>scaffold316|size347727_5832</td><td>G</td><td>A</td><td>intergenic_region</td><td>0.801346801</td><td>0.784443924</td><td>0.906230512</td></tr>
<tr><td>scaffold3323|size193577.9</td><td>copper/zinc superoxide dismutase</td><td>scaffold3323|size193577_31887</td><td>A</td><td>G</td><td>upstream_gene_variant</td><td>0.233744403</td><td>0.180342571</td><td>0.133627451</td></tr>
<tr><td>scaffold3858|size141125.12</td><td>copper/zinc superoxide dismutase</td><td>scaffold3858|size141125_59795</td><td>G</td><td>T</td><td>intergenic_region</td><td>0.941745718</td><td>0.919595568</td><td>0.975282738</td></tr>
<tr><td>scaffold386|size423945.19</td><td>copper/zinc superoxide dismutase</td><td>scaffold386|size423945_260985</td><td>G</td><td>A</td><td>intergenic_region</td><td>0.90698248</td><td>0.906473041</td><td>0.932462145</td></tr>
<tr><td>scaffold386|size423945.19</td><td>copper/zinc superoxide dismutase</td><td>scaffold386|size423945_385248</td><td>C</td><td>T</td><td>intergenic_region</td><td>0.774021528</td><td>0.752785615</td><td>0.844186905</td></tr>
<tr><td>scaffold4103|size91226.4</td><td>copper/zinc superoxide dismutase</td><td>scaffold4103|size91226_37732</td><td>G</td><td>T</td><td>intergenic_region</td><td>0.933780319</td><td>0.882075481</td><td>0.929166667</td></tr>
<tr><td>scaffold4160|size113367.1</td><td>copper/zinc superoxide dismutase</td><td>scaffold4160|size113367_7518</td><td>G</td><td>T</td><td>intron_variant</td><td>0.819940106</td><td>0.82228505</td><td>0.743310553</td></tr>
<tr><td>scaffold417|size388363.30</td><td>copper/zinc superoxide dismutase</td><td>scaffold417|size388363_187193</td><td>C</td><td>T</td><td>intergenic_region</td><td>0.614294589</td><td>0.556605471</td><td>0.714697965</td></tr>
<tr><td>scaffold4324|size150199.11</td><td>copper/zinc superoxide dismutase</td><td>scaffold4324|size150199_84106</td><td>G</td><td>A</td><td>downstream_gene_variant</td><td>0.612625475</td><td>0.644326545</td><td>0.545435123</td></tr>
<tr><td>scaffold4324|size150199.11</td><td>copper/zinc superoxide dismutase</td><td>scaffold4324|size150199_92727</td><td>T</td><td>A</td><td>upstream_gene_variant</td><td>0.325857598</td><td>0.349778653</td><td>0.25234441</td></tr>
<tr><td>scaffold4485|size144309.11</td><td>copper/zinc superoxide dismutase</td><td>scaffold4485|size144309_77872</td><td>C</td><td>A</td><td>downstream_gene_variant</td><td>0.757525178</td><td>0.765239871</td><td>0.726021441</td></tr>
<tr><td>scaffold523|size284731.6</td><td>copper/zinc superoxide dismutase</td><td>scaffold523|size284731_54936</td><td>T</td><td>A</td><td>intergenic_region</td><td>0.86629141</td><td>0.891232175</td><td>0.966918318</td></tr>
<tr><td>scaffold5416|size239699.13</td><td>copper/zinc superoxide dismutase</td><td>scaffold5416|size239699_125178</td><td>T</td><td>C</td><td>upstream_gene_variant</td><td>0.49920093</td><td>0.496768713</td><td>0.435925209</td></tr>
<tr><td>scaffold562|size200826.1</td><td>copper/zinc superoxide dismutase</td><td>scaffold562|size200826_31587</td><td>G</td><td>C</td><td>intergenic_region</td><td>0.895978238</td><td>0.864448384</td><td>0.861020181</td></tr>
<tr><td>scaffold626|size237541.24</td><td>copper/zinc superoxide dismutase</td><td>scaffold626|size237541_124713</td><td>A</td><td>G</td><td>downstream_gene_variant</td><td>0.94443659</td><td>0.931910584</td><td>0.911318256</td></tr>
<tr><td>scaffold719|size199701.38</td><td>copper/zinc superoxide dismutase</td><td>scaffold719|size199701_125384</td><td>T</td><td>G</td><td>downstream_gene_variant</td><td>0.945979908</td><td>0.928118624</td><td>0.975097344</td></tr>
<tr><td>scaffold770|size231676.6</td><td>copper/zinc superoxide dismutase</td><td>scaffold770|size231676_26081</td><td>A</td><td>T</td><td>stop_gained</td><td>0.886541996</td><td>0.879853271</td><td>0.949438259</td></tr>
<tr><td>scaffold770|size231676.6</td><td>copper/zinc superoxide dismutase</td><td>scaffold770|size231676_26067</td><td>G</td><td>A</td><td>downstream_gene_variant</td><td>0.834662589</td><td>0.863033463</td><td>0.933245573</td></tr>
<tr><td>scaffold770|size231676.6</td><td>copper/zinc superoxide dismutase</td><td>scaffold770|size231676_47169</td><td>C</td><td>A</td><td>intergenic_region</td><td>0.839793719</td><td>0.847572362</td><td>0.901160729</td></tr>
<tr><td>scaffold770|size231676.6</td><td>copper/zinc superoxide dismutase</td><td>scaffold770|size231676_54689</td><td>G</td><td>C</td><td>intergenic_region</td><td>0.92232598</td><td>0.924161255</td><td>0.958811309</td></tr>
<tr><td>scaffold818|size379131.4</td><td>copper/zinc superoxide dismutase</td><td>scaffold818|size379131_13952</td><td>G</td><td>A</td><td>downstream_gene_variant</td><td>0.925645701</td><td>0.924290077</td><td>0.981148217</td></tr>
<tr><td>scaffold937|size181198.13</td><td>copper/zinc superoxide dismutase</td><td>scaffold937|size181198_131636</td><td>T</td><td>C</td><td>intron_variant</td><td>0.95094511</td><td>0.936696668</td><td>0.982189522</td></tr>
<tr><td>scaffold3263|size165871.16</td><td>cytochrome b5 reductase 4-like isoform X3</td><td>scaffold3263|size165871_73814</td><td>T</td><td>A</td><td>intergenic_region</td><td>0.695305632</td><td>0.729963288</td><td>0.608682439</td></tr>
<tr><td>scaffold417|size388363.26</td><td>cytochrome P450 2C8-like</td><td>scaffold417|size388363_126041</td><td>T</td><td>G</td><td>upstream_gene_variant</td><td>0.840381663</td><td>0.849121604</td><td>0.894167564</td></tr>
<tr><td>scaffold3435|size139597.33</td><td>cytochrome P450 3A11-like isoform X1</td><td>scaffold3435|size139597_105262</td><td>T</td><td>C</td><td>intron_variant</td><td>0.938724385</td><td>0.936388268</td><td>0.971267534</td></tr>
<tr><td>scaffold9123|size36889.1</td><td>cytochrome P450 3A11-like isoform X1</td><td>scaffold9123|size36889_30428</td><td>G</td><td>A</td><td>intron_variant</td><td>0.95271618</td><td>0.922247958</td><td>0.955017597</td></tr>
<tr><td>scaffold5925|size155237.27</td><td>cytochrome P450 4F8-like</td><td>scaffold5925|size155237_135045</td><td>C</td><td>T</td><td>intergenic_region</td><td>0.207410521</td><td>0.213662287</td><td>0.311173346</td></tr>
<tr><td>scaffold1749|size108746.29</td><td>hypoxanthine-guanine phosphoribosyltransferase</td><td>scaffold1749|size108746_82049</td><td>T</td><td>C</td><td>intron_variant</td><td>0.59450892</td><td>0.631951765</td><td>0.565311005</td></tr>
<tr><td>scaffold1749|size108746.29</td><td>hypoxanthine-guanine phosphoribosyltransferase</td><td>scaffold1749|size108746_74624</td><td>C</td><td>T</td><td>upstream_gene_variant</td><td>0.496719307</td><td>0.538671197</td><td>0.599848237</td></tr>
<tr><td>scaffold337|size296661.9</td><td>laccase-4-like isoform X1</td><td>scaffold337|size296661_26903</td><td>T</td><td>C</td><td>upstream_gene_variant</td><td>0.365793187</td><td>0.359614338</td><td>0.445983161</td></tr>
<tr><td>scaffold4044|size72409.3</td><td>Laccase-6</td><td>scaffold4044|size72409_7801</td><td>T</td><td>G</td><td>upstream_gene_variant</td><td>0.89961108</td><td>0.894926839</td><td>0.801538877</td></tr>
<tr><td>scaffold2334|size95770.2</td><td>tyrosinase B3.2</td><td>scaffold2334|size95770_50948</td><td>A</td><td>T</td><td>intergenic_region</td><td>0.928754309</td><td>0.916319428</td><td>0.870859528</td></tr>
<tr><td>scaffold2426|size145584.3</td><td>tyrosinase B4</td><td>scaffold2426|size145584_113288</td><td>C</td><td>T</td><td>intergenic_region</td><td>0.723050765</td><td>0.674079207</td><td>0.759529895</td></tr>
<tr><td>scaffold6020|size75420.4</td><td>tyrosinase B4</td><td>scaffold6020|size75420_14397</td><td>C</td><td>T</td><td>downstream_gene_variant</td><td>0.547817696</td><td>0.554753935</td><td>0.509860859</td></tr>
<tr><td>scaffold5775|size102228.1</td><td>tyrosinase BPmax1</td><td>scaffold5775|size102228_18945</td><td>G</td><td>A</td><td>intergenic_region</td><td>0.927516234</td><td>0.929495074</td><td>1</td></tr>
<tr><td>scaffold5775|size102228.1</td><td>tyrosinase BPmax1</td><td>scaffold5775|size102228_21643</td><td>G</td><td>A</td><td>intergenic_region</td><td>0.936159694</td><td>0.947860963</td><td>1</td></tr>
<tr><td>scaffold1797|size279199.10</td><td>tyrosinase-like protein 1</td><td>scaffold1797|size279199_140999</td><td>C</td><td>A</td><td>intergenic_region</td><td>0.276081974</td><td>0.357726257</td><td>0.443553626</td></tr>
<tr><td>scaffold1797|size279199.10</td><td>tyrosinase-like protein 1</td><td>scaffold1797|size279199_75418</td><td>A</td><td>G</td><td>intergenic_region</td><td>0.678819444</td><td>0.674577482</td><td>0.572349629</td></tr>
<tr><td>scaffold1797|size279199.10</td><td>tyrosinase-like protein 1</td><td>scaffold1797|size279199_8228</td><td>A</td><td>G</td><td>intergenic_region</td><td>0.2434133</td><td>0.240490548</td><td>0.17905931</td></tr>
<tr><td>scaffold9953|size33018.5</td><td>tyrosinase-like protein 1</td><td>scaffold9953|size33018_3342</td><td>G</td><td>A</td><td>intergenic_region</td><td>0.718320118</td><td>0.796330694</td><td>0.801337166</td></tr>
<tr><td>scaffold10826|size62665.3</td><td>tyrosine-protein kinase PR2 isoform X1</td><td>scaffold10826|size62665_1539</td><td>A</td><td>G</td><td>downstream_gene_variant</td><td>0.241963947</td><td>0.208215498</td><td>0.39748847</td></tr>
<tr><td>scaffold10826|size62665.3</td><td>tyrosine-protein kinase PR2 isoform X1</td><td>scaffold10826|size62665_1608</td><td>G</td><td>A</td><td>downstream_gene_variant</td><td>0.214041975</td><td>0.19375126</td><td>0.305384199</td></tr>
<tr><td>scaffold1018|size159565.26</td><td>tyrosine-protein kinase transmembrane receptor Ror</td><td>scaffold1018|size159565_123029</td><td>A</td><td>T</td><td>intron_variant</td><td>0.772203314</td><td>0.789272234</td><td>0.879810161</td></tr>
<tr><td>scaffold6110|size54108.10</td><td>Tyrosine-protein phosphatase non-receptor type 1</td><td>scaffold6110|size54108_21921</td><td>C</td><td>T</td><td>downstream_gene_variant</td><td>0.744289773</td><td>0.748952037</td><td>0.752568394</td></tr>
<tr><td>scaffold5286|size98312.12</td><td>tyrosine-protein phosphatase non-receptor type 11 isoform X1</td><td>scaffold5286|size98312_75622</td><td>T</td><td>A</td><td>intron_variant</td><td>0.93797954</td><td>0.909285714</td><td>0.976388889</td></tr>
<tr><td>scaffold4726|size85232.4</td><td>tyrosine-protein phosphatase non-receptor type 23</td><td>scaffold4726|size85232_2882</td><td>C</td><td>T</td><td>intergenic_region</td><td>0.880592385</td><td>0.890455305</td><td>0.885397942</td></tr>
<tr><td>scaffold4726|size85232.4</td><td>tyrosine-protein phosphatase non-receptor type 23</td><td>scaffold4726|size85232_63146</td><td>T</td><td>G</td><td>intergenic_region</td><td>0.825057296</td><td>0.829744542</td><td>0.849187874</td></tr>
<tr><td>scaffold1490|size115539.4</td><td>visual pigment-like receptor peropsin</td><td>scaffold1490|size115539_64221</td><td>T</td><td>A</td><td>intergenic_region</td><td>0.83968423</td><td>0.85218254</td><td>0.922148655</td></tr>
<tr><td>scaffold5047|size126357.7</td><td>Vitamin D(3) 25-hydroxylase</td><td>scaffold5047|size126357_6322</td><td>C</td><td>A</td><td>upstream_gene_variant</td><td>0.623292643</td><td>0.63032377</td><td>0.802138113</td></tr>
<tr><td>scaffold5976|size55229.3</td><td>Xanthine dehydrogenase/oxidase</td><td>scaffold5976|size55229_32252</td><td>A</td><td>G</td><td>downstream_gene_variant</td><td>0.811070962</td><td>0.808213334</td><td>0.881810338</td></tr>
</table>

C - List of all green specific SNPs, from left to right: with the transcripts code; the proteins that are encoding by genes related to pigmentation impacted by the SNPs; the SNP place corresponding to the number of base (after the underscore) in the scaffold code; reference allele: alternative allele; impact of the SNP; and allelic frequencies for red, yellow and green phenotypes.

<table>
<tr><th>transcripts</th><th>Gene name</th><th>SNP</th><th>R</th><th>A</th><th>impact</th><th>Red's AF</th><th>Yellow's AF</th><th>Green's AF</th></tr>
<tr><td>scaffold605|size173110.34</td><td>**Cadherin EGF LAG seven-pass G-type receptor 2**</td><td>scaffold605|size173110_154272</td><td>G</td><td>A</td><td>downstream_gene_variant</td><td>0.805907087</td><td>0.777020225</td><td>0.789821316</td></tr>
<tr><td>scaffold910|size252188.4</td><td>**Cadherin-23**</td><td>scaffold910|size252188_14555</td><td>T</td><td>C</td><td>intron_variant</td><td>0.936815709</td><td>0.954407794</td><td>0.95638941</td></tr>
<tr><td>scaffold234|size272526.38</td><td>**cadherin-23-like isoform X3**</td><td>scaffold234|size272526_211612</td><td>C</td><td>T</td><td>intergenic_region</td><td>0.852221135</td><td>0.850536729</td><td>0.82749144</td></tr>
<tr><td>scaffold2505|size92724.1</td><td>**cadherin-23-like isoform X3**</td><td>scaffold2505|size92724_18810</td><td>T</td><td>C</td><td>intron_variant</td><td>0.86537074</td><td>0.865843672</td><td>0.90107426</td></tr>
<tr><td>scaffold14161|size18149.1</td><td>**calcium-binding protein LPS1-alpha-like**</td><td>scaffold14161|size18149_12593</td><td>A</td><td>T</td><td>intron_variant</td><td>0.836027236</td><td>0.824552687</td><td>0.817016807</td></tr>
<tr><td>scaffold791|size153329.12</td><td>**Cytochrome P450 20A1**</td><td>scaffold791|size153329_87835</td><td>T</td><td>C</td><td>intron_variant</td><td>0.691928077</td><td>0.698565283</td><td>0.712101868</td></tr>
<tr><td>scaffold791|size153329.12</td><td>**Cytochrome P450 20A1**</td><td>scaffold791|size153329_88867</td><td>G</td><td>T</td><td>intron_variant</td><td>0.805654401</td><td>0.840786398</td><td>0.81093886</td></tr>
<tr><td>scaffold1310|size120650.23</td><td>**cytochrome P450 2C8**</td><td>scaffold1310|size120650_80870</td><td>C</td><td>A</td><td>intron_variant</td><td>0.77810725</td><td>0.740362147</td><td>0.754549077</td></tr>
<tr><td>scaffold49|size265340.7</td><td>**cytochrome P450 2C8**</td><td>scaffold49|size265340_51856</td><td>C</td><td>A</td><td>upstream_gene_variant</td><td>0.670114386</td><td>0.750633017</td><td>0.753257132</td></tr>
<tr><td>scaffold49|size265340.7</td><td>**cytochrome P450 2C8**</td><td>scaffold49|size265340_61554</td><td>C</td><td>T</td><td>intron_variant</td><td>0.888403396</td><td>0.898256154</td><td>0.89336054</td></tr>
<tr><td>scaffold49|size265340.6</td><td>**cytochrome P450 2C8-like**</td><td>scaffold49|size265340_38705</td><td>A</td><td>C</td><td>intron_variant</td><td>0.889979989</td><td>0.952203486</td><td>0.966111111</td></tr>
<tr><td>scaffold1341|size119781.4</td><td>**Cytochrome P450 4A25**</td><td>scaffold1341|size119781_6168</td><td>C</td><td>T</td><td>downstream_gene_variant</td><td>0.918895657</td><td>0.949343501</td><td>0.957331175</td></tr>
<tr><td>scaffold1676|size364098.104</td><td>**cytochrome P450 4F12-like**</td><td>scaffold1676|size364098_330215</td><td>A</td><td>T</td><td>intergenic_region</td><td>0.920481336</td><td>0.934329138</td><td>0.906390929</td></tr>
<tr><td>scaffold1676|size364098.104</td><td>**cytochrome P450 4F12-like**</td><td>scaffold1676|size364098_331860</td><td>C</td><td>A</td><td>intergenic_region</td><td>0.943708073</td><td>0.95677454</td><td>0.944660203</td></tr>
<tr><td>scaffold551|size158850.4</td><td>**ferritin 1**</td><td>scaffold551|size158850_20422</td><td>A</td><td>C</td><td>upstream_gene_variant</td><td>0.940316116</td><td>0.969825621</td><td>0.951635209</td></tr>
<tr><td>scaffold5249|size131904.15</td><td>**ferrochelatase, mitochondrial**</td><td>scaffold5249|size131904_104547</td><td>G</td><td>A</td><td>intergenic_region</td><td>0.926883141</td><td>0.934336563</td><td>0.9367971</td></tr>
<tr><td>scaffold2158|size160354.18</td><td>**flavin reductase (NADPH)**</td><td>scaffold2158|size160354_118513</td><td>T</td><td>C</td><td>intergenic_region</td><td>0.9193817</td><td>0.898537274</td><td>0.890424598</td></tr>
<tr><td>scaffold1115|size353168.19</td><td>**glutathione S-transferase 1-like**</td><td>scaffold1115|size353168_63863</td><td>A</td><td>T</td><td>intron_variant</td><td>0.270505546</td><td>0.250070173</td><td>0.228927432</td></tr>
<tr><td>scaffold12296|size49253.2</td><td>**multidrug resistance-associated protein 1**</td><td>scaffold12296|size49253_11119</td><td>A</td><td>G</td><td>downstream_gene_variant</td><td>0.800833126</td><td>0.742559479</td><td>0.771712548</td></tr>
<tr><td>scaffold915|size243652.70</td><td>**Multidrug resistance-associated protein 1**</td><td>scaffold915|size243652_171213</td><td>C</td><td>G</td><td>upstream_gene_variant</td><td>0.868952339</td><td>0.898203015</td><td>0.912329655</td></tr>
<tr><td>scaffold2357|size95418.10</td><td>**multidrug resistance-associated protein 1 isoform X1**</td><td>scaffold2357|size95418_83886</td><td>C</td><td>A</td><td>intergenic_region</td><td>0.830724746</td><td>0.887748418</td><td>0.855921053</td></tr>
<tr><td>scaffold7087|size47719.1</td><td>**multidrug resistance-associated protein 1-like isoform X1**</td><td>scaffold7087|size47719_1109</td><td>A</td><td>C</td><td>intergenic_region</td><td>0.934650909</td><td>0.979166667</td><td>0.972170686</td></tr>
<tr><td>scaffold7087|size47719.1</td><td>**multidrug resistance-associated protein 1-like isoform X1**</td><td>scaffold7087|size47719_1241</td><td>A</td><td>T</td><td>intergenic_region</td><td>0.860992851</td><td>0.870014153</td><td>0.866125601</td></tr>
<tr><td>scaffold11594|size51006.5</td><td>**perlucin-like protein**</td><td>scaffold11594|size51006_34205</td><td>A</td><td>T</td><td>upstream_gene_variant</td><td>0.841733661</td><td>0.897606122</td><td>0.923381611</td></tr>
<tr><td>scaffold3547|size129194.18</td><td>**perlucin-like protein**</td><td>scaffold3547|size129194_105367</td><td>A</td><td>T</td><td>downstream_gene_variant</td><td>0.457122894</td><td>0.492490287</td><td>0.557910277</td></tr>
<tr><td>scaffold3547|size129194.18</td><td>**perlucin-like protein**</td><td>scaffold3547|size129194_105379</td><td>A</td><td>T</td><td>downstream_gene_variant</td><td>0.48554368</td><td>0.510598158</td><td>0.50144815</td></tr>
<tr><td>scaffold1567|size137386.7</td><td>**perlucin-like protein isoform X1**</td><td>scaffold1567|size137386_22342</td><td>G</td><td>T</td><td>upstream_gene_variant</td><td>0.275866069</td><td>0.259850464</td><td>0.32703608</td></tr>
<tr><td>scaffold454|size172198.10</td><td>**Tyrosinase 1**</td><td>scaffold454|size172198_160132</td><td>T</td><td>C</td><td>intergenic_region</td><td>0.78410398</td><td>0.867416285</td><td>0.870616152</td></tr>
<tr><td>scaffold454|size172198.10</td><td>**Tyrosinase 1**</td><td>scaffold454|size172198_56577</td><td>C</td><td>A</td><td>intergenic_region</td><td>0.919871795</td><td>0.926869472</td><td>0.936012748</td></tr>
<tr><td>scaffold1980|size291657.3</td><td>**tyrosinase B2.1**</td><td>scaffold1980|size291657_59414</td><td>C</td><td>G</td><td>intron_variant</td><td>0.950615033</td><td>0.967797443</td><td>0.946989114</td></tr>
<tr><td>scaffold101|size233448.23</td><td>**urease subunit alpha-like**</td><td>scaffold101|size233448_106064</td><td>A</td><td>G</td><td>intron_variant</td><td>0.359974162</td><td>0.359209226</td><td>0.429068463</td></tr>
<tr><td>scaffold101|size233448.23</td><td>**urease subunit alpha-like**</td><td>scaffold101|size233448_148567</td><td>G</td><td>A</td><td>intergenic_region</td><td>0.877671023</td><td>0.910906695</td><td>0.914858458</td></tr>
<tr><td>scaffold2909|size388074.62</td><td>**visual pigment-like receptor peropsin**</td><td>scaffold2909|size388074_257210</td><td>T</td><td>A</td><td>downstream_gene_variant</td><td>0.861870445</td><td>0.848308763</td><td>0.900331821</td></tr>
</table>

File S5:

A – Secondary structure prediction by I-Tasser for the PBGD reference sequence.

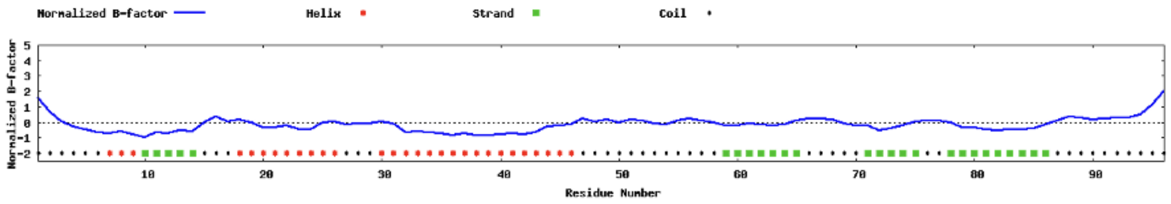

B – Secondary structure prediction by I-Tasser for the PBGD alternative sequence.

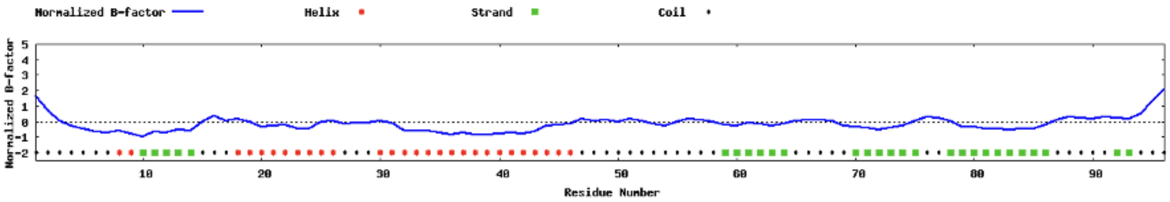

C – Secondary structure prediction by I-Tasser for the Shematrin reference sequence.

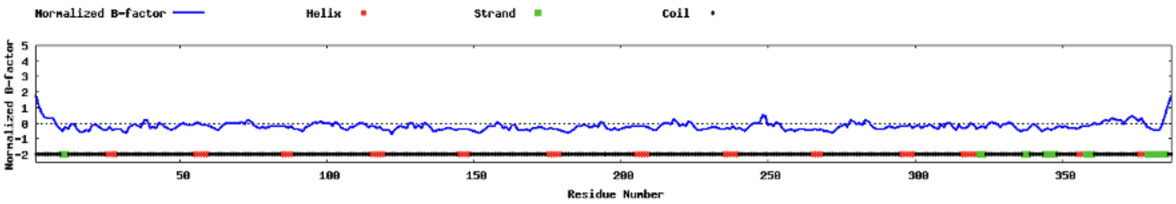

D – Secondary structure prediction by I-Tasser for the Shematrin alternative sequence.

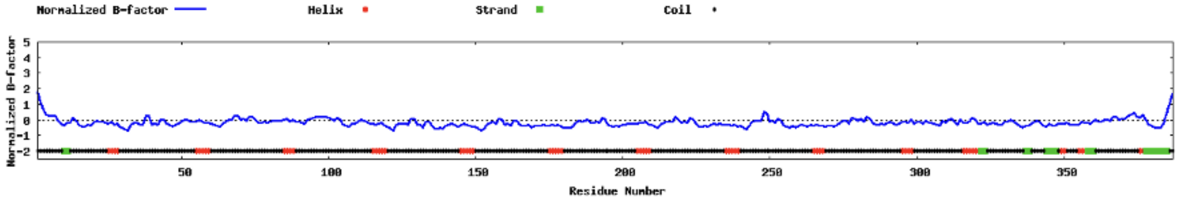

E – Secondary structure prediction by I-Tasser for the Cytochrome P450 3A29 reference sequence.

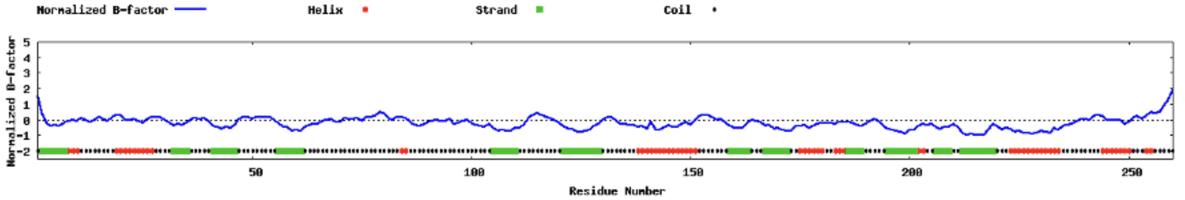

F – Secondary structure prediction by I-Tasser for the Cytochrome P450 3A29 alternative sequence.

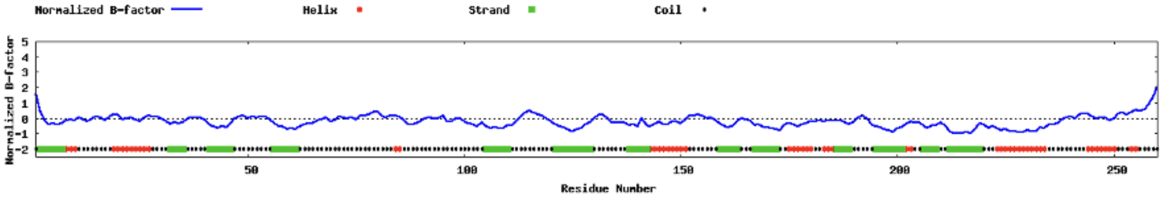

G – Secondary structure prediction by I-Tasser for the Cadherin reference sequence.

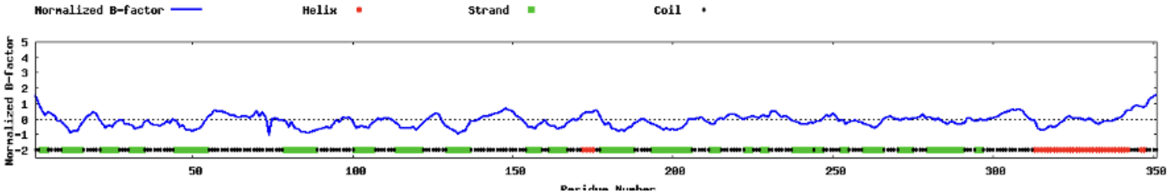

H – Secondary structure prediction by I-Tasser for the Cadherin alternative sequence.

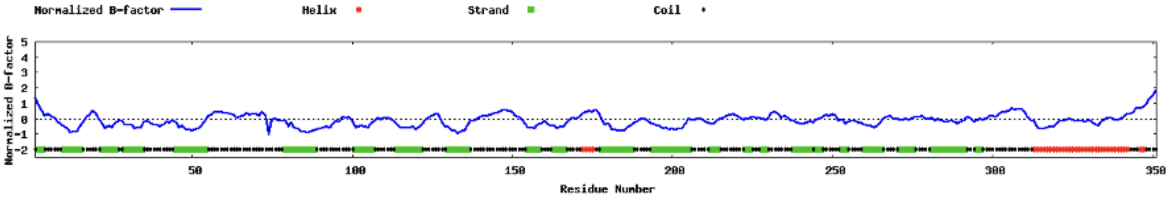

Supplement: Supplementary file 1 — Appendix S1 [file EVA-16-408-s001.zip › EVA_13464_Supplementary_files_S02_to_S05.pdf]
